# Exosomal miR-122-5p from tubular cells ameliorates renal interstitial fibrosis by regulating fibroblasts via HIF-1α

**DOI:** 10.1038/s41420-025-02739-8

**Published:** 2025-10-21

**Authors:** Jifeng Yang, Fengxia Bai, Yao Li, Qian Gao, Linqian Li, Zhaomu Zeng, Juan Jia, Nan Jia, Yuhao Zhang, Yan Gao, Haisong Zhang

**Affiliations:** 1 School of Clinical Medicine, Hebei University, Affiliated Hospital of Hebei University, Baoding, China; 2 Hebei Provincial Key Laboratory of Skeletal Metabolic Physiology of Chronic Kidney Disease, Affiliated Hospital of Hebei University, Baoding, China; 3 Department of Nephrology, The Affiliated Hospital of Yangzhou University, Yangzhou University, Yangzhou, China; 4 Basic Research Key Laboratory of General Surgery for Digital Medicine, Baoding, China; 5 3D Image and 3D Printing Center, Affiliated Hospital of Hebei University, Baoding, China; 6 Department of Neurosurgery, Jiangxi Provincial People’s Hospital, The First Affiliated Hospital of Nanchang Medical College, Nanchang, China; 7 Department of Neurology, Affiliated Hospital of Hebei University, Baoding, China; 8 Cancer Center, Department of Neurosurgery, Zhejiang Provincial People’s Hospital (Affiliated People’s Hospital), Hangzhou Medical College, Hangzhou, Zhejiang China

**Keywords:** Renal fibrosis, Translational research

## Abstract

Renal interstitial fibrosis (RIF) is a common pathological feature and final manifestation of chronic progressive kidney disease. Although exosome-mediated intercellular communication is known to play a crucial role in RIF, the mechanism by which injured tubular epithelial cells (TECs) contribute to fibrogenesis remains incompletely understood. In this study, we investigated the role of exosomal miR-122-5p derived from TECs in modulating fibroblast activation and renal fibrosis. Exosomes were isolated from kidneys of unilateral ureteral obstruction (UUO) mice and from TGF-β1-stimulated HK-2 cells. These exosomes were either co-cultured with fibroblasts (NRK-49F cells) or injected into UUO mice via the tail vein. High-throughput miRNA profiling was used to identify differentially expressed miRNAs in exosomes derived from HK-2 cells, and miR-122-5p was selected for further investigation based on expression level and bioinformatic prediction. Functional analyses were performed using miRNA mimics, inhibitors, and target validation assays. The results showed that exosomal miR-122-5p was significantly downregulated in both fibrotic kidneys and TGF-β1-induced HK-2 cells. In vivo and in vitro, restoration of miR-122-5p levels markedly attenuated renal fibrosis, as evidenced by the reduction of fibrotic markers including α-smooth muscle actin, fibronectin, and collagen I. Mechanistically, miR-122-5p was found to directly target hypoxia-inducible factor 1-alpha (HIF-1α), thereby inhibiting activation of the TGF-β1/Smad signaling pathway. These findings suggest that decreased expression of miR-122-5p in TEC-derived exosomes promotes renal fibrosis through the HIF-1α/TGF-β1/Smad axis, while reintroduction of miR-122-5p can effectively reverse these effects. Taken together, our study provides new insights into the intercellular communication between TECs and fibroblasts via exosomal miRNAs, and identifies miR-122-5p as a potential therapeutic target for the treatment of renal interstitial fibrosis.

## Introduction

Chronic kidney disease (CKD) is a significant public health problem that affects approximately 10–14% of the global population, with persistently high rates of morbidity and mortality [1–3]. Renal interstitial fibrosis (RIF) is a common pathway in the progression of CKD and a key research focus in kidney fibrosis [4]. RIF refers to the transformation of renal intrinsic cells in response to pathogenic stimuli, under the influence of fibrogenic factors, cytokines, and growth factors, leading to changes in cell phenotype, the loss of normal nephrons, and replacement with proliferating fibroblasts and myofibroblasts. This process results in the production and accumulation of extracellular matrix components, including collagen fibers and fibronectin, thus leading to tubulointerstitial fibrosis (TIF) and, ultimately, the loss of kidney function [5, 6]. Epithelial-mesenchymal transition (EMT) is a major pathological process that can promote RIF. The tubular interstitial interaction initiated by damaged renal tubules is a core driver of CKD progression [7, 8]. Therefore, identifying the specific molecular mechanisms that underlie RIF is beneficial for mitigating the progression of CKD [9].

Exosomes are vesicles surrounded by a lipid bilayer with a diameter of 30–100 nm that are derived from intracellular multivesicular bodies. These small vesicles are produced by various types of cells under both physiological and pathological conditions and are present in the extracellular space and all body fluids [10, 11]. Exosomes are a variety of signaling molecules, including microRNAs, long non-coding RNAs, mRNAs, proteins, and lipids [12]. In addition, exosomes are able to shuttle between cells to deliver biological information and regulate recipient cells, thereby mediating intercellular communication [13]. The renal tubular epithelium is considered a primary target and central focus of kidney injury. A large body of evidence now indicates that stimulated tubular cells can communicate with glomerular cells, interstitial fibroblasts, macrophages, and other cell types *via* exosomes to trigger a range of cellular responses [14–16]. Thus, exosomes play an important role in transmitting information that enables specific and efficient intercellular communication.

MicroRNAs (miRNAs) are a class of endogenous small non-coding RNAs that negatively regulate the expression of target genes by binding to the 3′-untranslated region (UTR) of mRNAs [17]. An increasing body of evidence suggests that changes in the expression of miRNAs within exosomes derived from renal tubules are closely related to the occurrence and progression of RIF. For example, in a mouse model of unilateral ureteral obstruction (UUO), the presence of miR-21 in exosomes derived from tubular epithelial cells activated fibroblasts *via* the PTEN/Akt pathway, thus accelerating the progression of fibrosis in the obstructed kidney [18]. In a rat model of unilateral ischemia-reperfusion injury (UIRI), the presence of miR-150-5p in exosomes derived from tubular cells was shown to mediate tubular epithelial cell-fibroblast communication, negatively regulating the expression of suppressor of cytokine signaling 1, thereby activating fibroblasts and promoting the progression of renal fibrosis [19].

When caused by ischemia-reperfusion (IR), renal injury is often accompanied by irreversible renal fibrosis. In the IR-induced renal injury model, tubular epithelial cells have been shown to secrete exosomes containing miR-150, which was shown to initiate the activation and proliferation of fibroblasts during kidney repair, thereby promoting the progression of renal fibrosis [19]. Experimental studies in a mouse model of diabetic kidney disease (DKD) demonstrated a clear reduction in the secretion of exosomes isolated and purified from the renal cortex and proximal tubular cells when treated with high-glucose. Furthermore, the levels of Eno1 were correlated with the progression of disease [20]. In addition, exosomes may participate in the paracrine signaling mechanism involved in DKD RIF by regulating the activation and proliferation of fibroblasts [21]. In the study, we posited that exosomal miRNA released by TECs could initiate TIF through conveying the ameliorative signals to activate fibroblasts. These observations highlight the fact that exosomes may serve as a unique and robust vehicle for intercellular signal exchange during the pathogenesis of CKD. In this context, it is important to investigate the potential role of exosomes in the pathological process of tubular fibrosis. Our findings highlight the importance of exosomes in the pathogenesis of RIF and suggest new avenues for developing CKD therapies.

## Results

### TGF-β1-induced fibrosis in HK-2 cells

As a major pro-fibrotic factor, TGF-β1 exerts a primary stimulatory effect in the pathological process responsible for RIF. At the cellular level, we incubated HK-2 cells in exosome-free serum medium with or without TGF-β1 (final concentration: 10 ng/mL) for 24 h to investigate the state of damage/stress in renal tubular epithelial cells. Light microscopy revealed that cells in the NC group were tightly connected and appeared cobblestone-like, while in the TGF-β1 group, some cells had detached from their surrounding cells and appeared spindle-shaped (Fig. S1A). Compared to the NC group, western blotting confirmed that the TGF-β1 group showed a significant increase in α-SMA protein expression (*P* < 0.01) and a significant decrease in E-cadherin protein expression (*P* < 0.01), indicating that the cell model had been successfully constructed (Fig. S1B–D).

### Establishment of a mouse model of UUO

At the animal level, we used the classic UUO method to construct a mouse model of RIF. Analysis revealed that in the Sham group, the kidneys of mice were of normal size (Fig. S2A), with a clear glomerular structure and tightly arranged renal tubules under HE staining; no inflammatory cell infiltration was observed. Masson’s and Sirius Red (SR) staining revealed no evidence of fibrosis or collagen fiber deposition. In the UUO group, the kidneys of mice were enlarged. HE staining revealed vacuolar degeneration in the tubular epithelial cells, compensatory tubular dilation, and inflammatory cell infiltration in the interstitium. Masson’s and SR staining revealed broken tubular basement membranes, interstitial collagen deposition, and increased fibrosis (Fig. S2B). Compared to the Sham group, western blotting revealed that the UUO group had significantly increased α-SMA protein expression (*P* < 0.01) and decreased E-cadherin protein expression in kidney tissue (*P* < 0.01), along with significantly increased serum creatinine and blood urea nitrogen levels (*P* < 0.01), thus indicating successful construction of the animal model (Fig. S2C–J).

### Isolation, characterization, distribution, and uptake of HK-2 cell-derived exosomes in tubular-fibroblast communication

Based on preliminary experimental results and an extensive literature review, we hypothesized that exosomes may play a role in tubular-fibroblast communication. We then proceeded with the isolation and identification of exosomes from HK-2 cells. TEM analysis revealed that the exosomes adopted a typical cup-shaped structure (Fig. S3A). Nanoparticle tracking analysis indicated that most particle diameters ranged between 40 nm and 140 nm, with an average exosome particle diameter of 59.98 ± 9.88 nm (Fig. S3B). Western blot analysis confirmed the expression of positive protein markers (TSG-101 and CD63), with no expression of HSP70 and Calnexin. (CL represents the cell lysate sample, used as a positive control in exosome Western blot identification) (Fig. S3C).

At the cellular level, fluorescence microscopy revealed significant PKH67 expression in NRK-49F cells, indicating that these cells were able to take up exosomes secreted by HK-2 cells (Fig. S3D). At the animal level, in vivo imaging of small animals showed that after the tail vein injection of exosomes from the TGF-β1 group, the fluorescence intensity was more concentrated in the kidney tissue of UUO mice, suggesting that kidney injury enhances the uptake capacity for exosomes, demonstrating the homing-like behavior of exosomes (Fig. S3E).

### The miRNA expression profile of exosomes in HK-2 cells

**Fig. 1:**
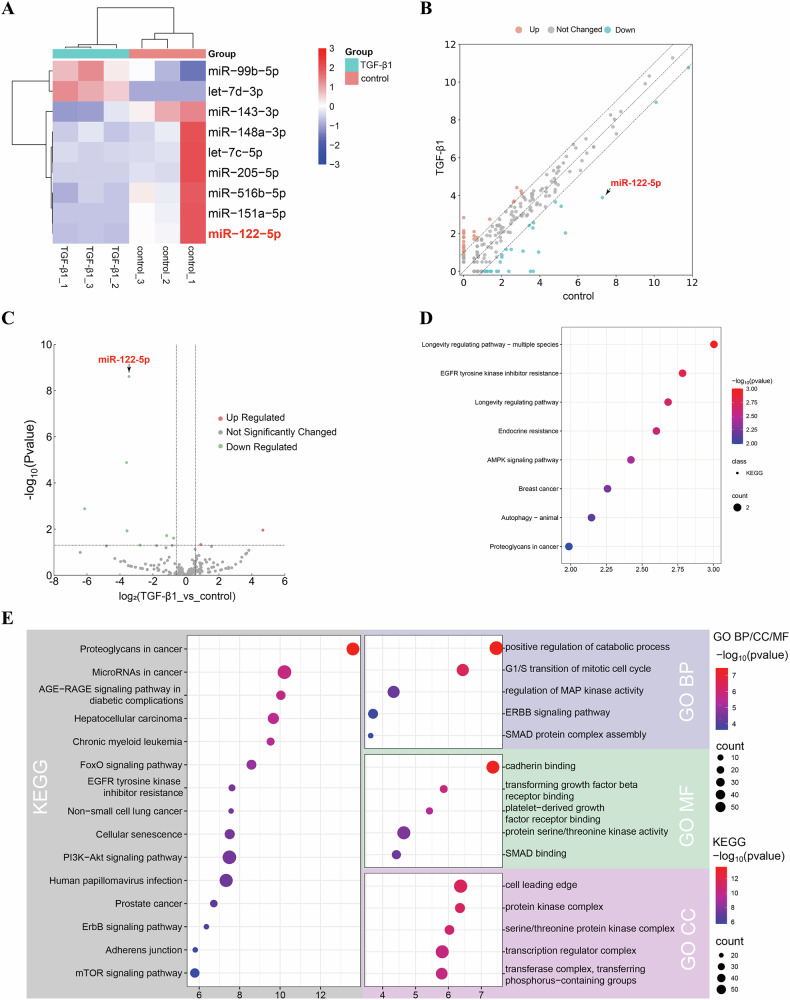
The miRNA expression profile of exosomes in HK-2 cells. **A** Cluster plot of miRNA expression derived from HK-2 cell exosomes; **B** Scatter plot of miRNA expression derived from HK-2 cell exosomes; **C** Volcano plot of miRNA expression derived from HK-2 cell exosomes; **D** The results obtained by KEGG pathway analysis; **E** The results obtained by Gene Ontology (GO) functional enrichment analysis.

Next, we determined the miRNA signature of HK-2 cell exosomes using the DESeq2 package (Estimate variance-mean dependence in count data from high-throughput sequencing assays and test for differential expression based on a model using the negative binomial distribution). Exosomal miRNA expression was significantly different in HK-2 cells with and without TGF-β1 treatment, with a fold change of 1.5 and *p* value of 0.05. Hierarchical clustering analysis of miRNA expression found that nine miRNAs (two up-regulated and seven down-regulated) were differentially expressed (Fig. 1A–C). We performed KEGG pathway and Gene Ontology (GO) function enrichment for the up-regulated and down-regulated miRNAs (Fig. 1D, E). miRNA analysis showed that the target genes associated with up-regulated miRNAs were significantly enriched in multiple biological pathways related to cancer, longevity, drug resistance and signaling pathways, but not related to renal fibrosis. miRNA analysis also revealed that the target genes of down-regulated miRNAs were significantly enriched in multiple pathways and functions related to cancer, signaling pathways, metabolic regulation and cell structure. KEGG and GO enrichment analyses provided a basis for investigating the potential roles of these miRNAs in cell biological processes, suggesting that they may perform important regulatory roles in cancer and cell signaling regulation. These pathways may play an important role in the regulatory network of miRNAs to influence the biological effects of TGF-β1 treatment. Next, in order to investigate whether exosomes have the ability to regulate RIF, we searched the literature and finally selected miR-122-5p as the miRNA with the most obvious differential expression for further investigation.

### Exosomes secreted by HK-2 cells overexpressing miR-122-5p were able to improve fibrosis in NRK-49F cells induced by TGF-β1

**Fig. 2:**
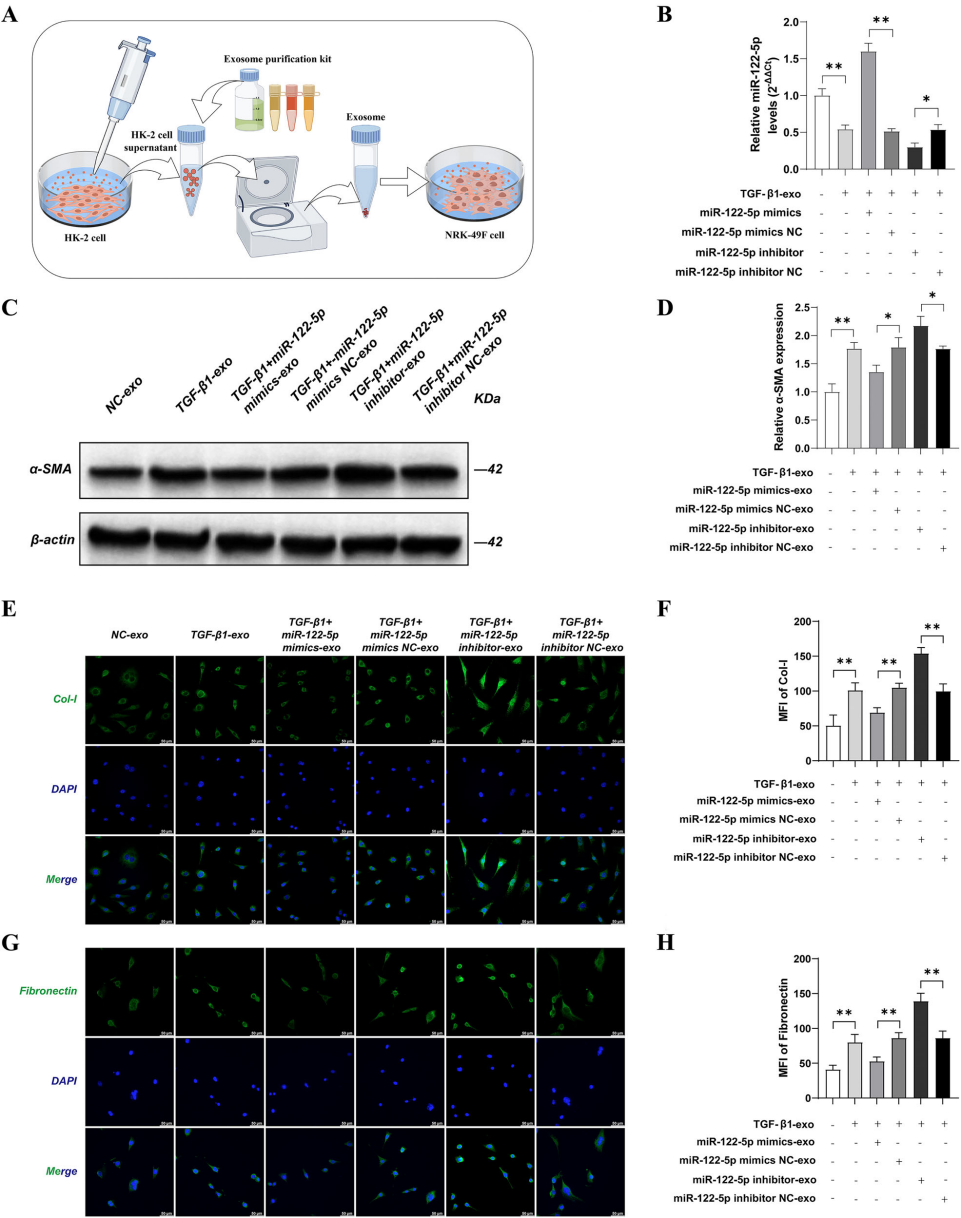
Exosomes secreted by HK-2 cells overexpressing miR-122-5p were able to improve fibrosis in NRK-49F cells induced by TGF-β1. **A** Flow diagram of exosomes co-cultured with NRK-49F cells stimulated by TGF-β1; **B** The levels of miR-122-5p miRNA in the exosomes obtained from HK-2 cells were detected by quantitative polymerase chain reaction (qPCR); **C** Western blot was used to detect the expression of α-SMA in each group; **D** Relative expression levels of α-SMA in each group; **E**, **F** The detection of Col-I expression in NRK-49F cells were conducted by immunofluorescence. **G**, **H** The detection of Fibronectin expression in NRK-49F cells were conducted by immunofluorescence. Results are shown as mean ± SD for three individual experiments. ^**^P < 0.01.

At the cellular level, we first overexpressed and knocked-down miR-122-5p in HK-2 cells and then co-incubated extracted exosomes with NRK-49F cells stimulated with TGF-β1 (Fig. 2A). First, we measured the levels of miR-122-5p in exosomes from each group by qRT-PCR to verify transfection efficiency. Compared with the NC group, miR-122-5p expression in exosomes from TGF-β1-treated HK-2 cells was significantly reduced (*P* < 0.01). Compared with the TGF-β1 group, there was no significant change in miR-122-5p expression in exosomes from the TGF-β1 + miR-122-5p mimic NC and TGF-β1 + miR-122-5p inhibitor NC groups (*P* > 0.05). However, compared with the TGF-β1 + miR-122-5p mimic NC group, miR-122-5p expression was significantly increased in the TGF-β1 + miR-122-5p mimic group (*P* < 0.01), and compared with the TGF-β1 + miR-122-5p inhibitor NC group, miR-122-5p expression was significantly decreased in the TGF-β1 + miR-122-5p inhibitor group in HK-2 cell exosomes (*P* < 0.01) (Fig. 2B).

Next, to investigate whether exosomes are able to regulate RIF, we reviewed the relevant literature and selected miR-122-5p, which showed the most significant differential expression, for further investigation. Compared with the NC-exo group, western blotting revealed that the expression of α-SMA in NRK-49F cells was significantly increased in the TGF-β1-exo group (*P* < 0.01). Compared with the TGF-β1-exo group, there was no significant change in α-SMA expression in NRK-49F cells in the TGF-β1 + miR-122-5p mimic NC-exo and TGF-β1 + miR-122-5p inhibitor NC-exo groups (*P* > 0.05). However, compared with the TGF-β1 + miR-122-5p mimic NC-exo group, α-SMA expression was significantly reduced in the TGF-β1 + miR-122-5p mimic group in NRK-49F cells (*P* < 0.01). Furthermore, compared with the TGF-β1 + miR-122-5p inhibitor NC-exo group, the expression of α-SMA was significantly increased in the TGF-β1 + miR-122-5p inhibitor-exo group in NRK-49F cells (*P* < 0.01) (Fig. 2C, D).

Compared with the NC-exo group, immunofluorescence analysis showed that the expression levels of Col-I and Fibronectin were significantly increased in NRK-49F cells in the TGF-β1-exo group (*P* < 0.01). Compared with the TGF-β1-exo group, there was no significant change in the expression of Col-I and Fibronectin in NRK-49F cells in the TGF-β1 + miR-122-5p mimic NC-exo and TGF-β1 + miR-122-5p inhibitor NC-exo groups (*P* > 0.05). However, compared with the TGF-β1 + miR-122-5p mimic NC group, the expression of Col-I and Fibronectin was significantly reduced in the TGF-β1 + miR-122-5p mimic group in NRK-49F cells (*P* < 0.01), but significantly increased in the TGF-β1 + miR-122-5p inhibitor group compared with the TGF-β1 + miR-122-5p inhibitor NC group (*P* < 0.01). Collectively, these results indicated that exosomal miR-122-5p had the ability to regulate RIF at the cellular level (Fig. 2E–H).

### Effects of overexpressing or inhibiting miR-122-5p in exosomes on renal function and EMT markers in UUO mice

**Fig. 3:**
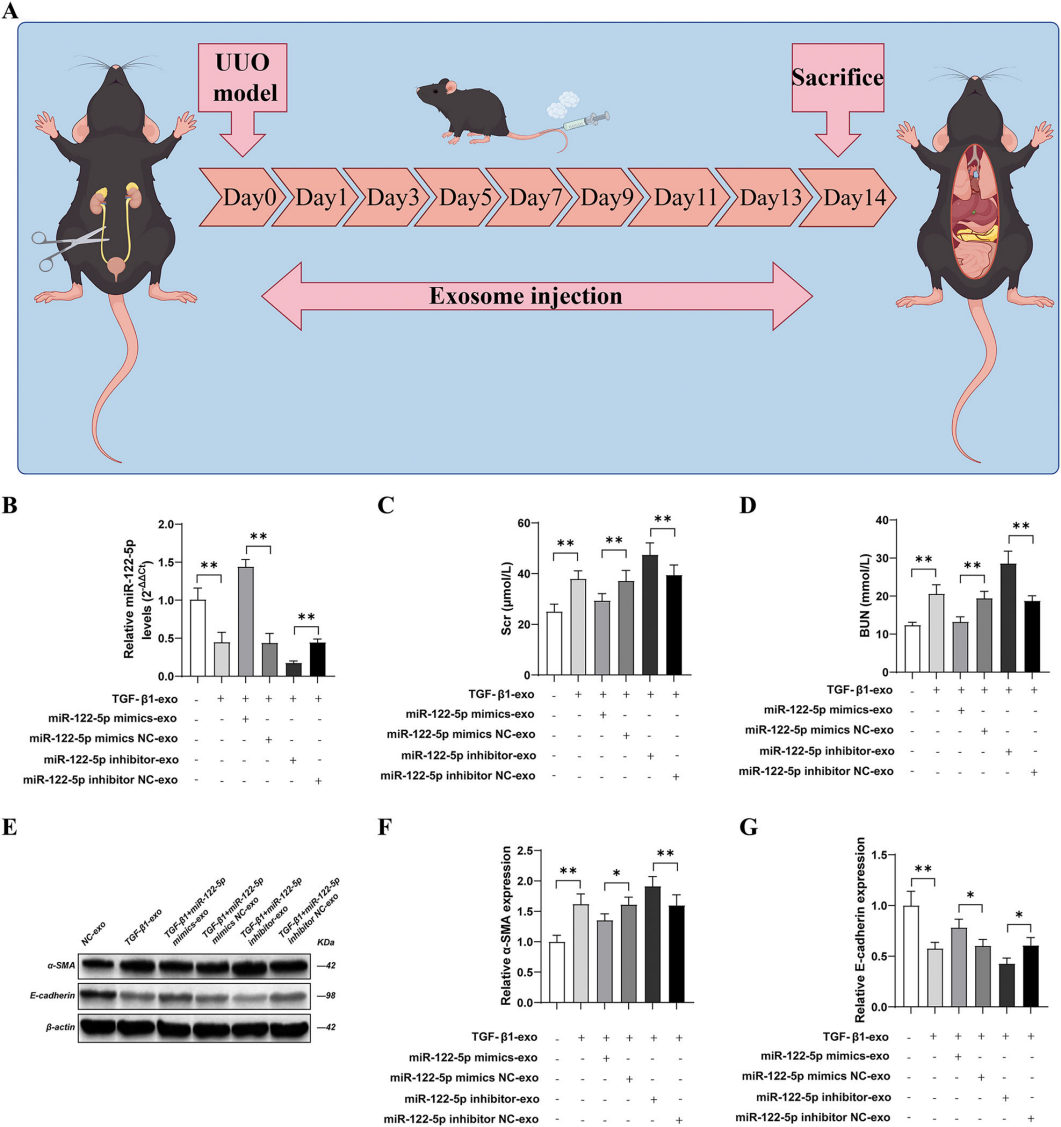
Effects of overexpressing or inhibiting miR-122-5p in Exosomes on renal function and EMT markers in UUO mice. **A** Flow diagram of exosomes injected into the tail vein of UUO mice; **B** The levels of miR-122-5p miRNA in kidney tissue were detected by quantitative polymerase chain reaction (qPCR); **C** The levels of creatinine in mice serum were determined by a biochemical analyzer; **D** The levels of blood urea nitrogen in mice serum were determined by a biochemical analyzer; **E** The expression levels of α-SMA and E-cadherin in kidney tissue were detected by western blotting; **F** Relative expression levels of α-SMA in renal tissues; **G**. Relative expression levels of E-cadherin in renal tissues. Results are shown as mean ± SD for six individual experiments. *P < 0.05, **P < 0.01.

At the animal level, we first established a UUO model in normal C57BL/6 mice. Then, exosomes containing either overexpressed or knocked-down miR-122-5p were injected via the tail vein, based on experimental requirements. Then, we collected samples of serum and kidney tissue from the mice in each group (Fig. 3A). qRT-PCR analysis showed that, compared with the NC-exo group, the levels of miR-122-5p miRNA in kidney tissue were significantly lower in the TGF-β1-exo group (*P* < 0.01); biochemical analysis further confirmed that the serum levels of creatinine and blood urea nitrogen were significantly increased (*P* < 0.01). Compared with the TGF-β1-exo group, there were no significant differences in the levels of miR-122-5p miRNA in kidney tissue or in the serum levels of creatinine and blood urea nitrogen in the TGF-β1 + miR-122-5p mimic NC-exo and TGF-β1 + miR-122-5p inhibitor NC-exo groups (*P* > 0.01). However, compared with the TGF-β1 + miR-122-5p mimic NC-exo group, the TGF-β1 + miR-122-5p mimic-exo group showed a significant increase in miR-122-5p miRNA levels in kidney tissue (*P* < 0.01) and a significant reduction in the serum levels of creatinine and blood urea nitrogen (*P* < 0.01). Conversely, compared with the TGF-β1 + miR-122-5p inhibitor NC-exo group, the TGF-β1 + miR-122-5p inhibitor-exo group showed a significant reduction in the levels of miR-122-5p miRNA in kidney tissue (*P* < 0.01) and a significant increase in the serum levels of creatinine and blood urea nitrogen (*P* < 0.01) (Fig. 3B–D).

Compared with the NC-exo group, western blotting revealed that the expression of α-SMA was significantly increased (*P* < 0.01) and E-cadherin expression was significantly decreased in kidney tissue from the TGF-β1-exo group (*P* < 0.01). Compared with the TGF-β1-exo group, there was no significant change in the expression of α-SMA in kidney tissue in the TGF-β1 + miR-122-5p mimic NC-exo and TGF-β1 + miR-122-5p inhibitor NC-exo groups (*P* > 0.01). However, compared with the TGF-β1 + miR-122-5p mimic NC-exo group, the expression of α-SMA was significantly reduced (*P* < 0.01) while the expression of E-cadherin was significantly increased (*P* < 0.01) in the TGF-β1 + miR-122-5p mimic-exo group in kidney tissue. Conversely, compared with the TGF-β1 + miR-122-5p inhibitor NC-exo group, the expression of α-SMA was significantly increased (*P* < 0.01) while the expression of E-cadherin was significantly reduced (*P* < 0.01) in the TGF-β1 + miR-122-5p inhibitor-exo group in kidney tissue (Fig. 3E–G).

### Effects of overexpressing or inhibiting miR-122-5p in exosomes on renal fibrosis and collagen deposition in UUO mice

**Fig. 4:**
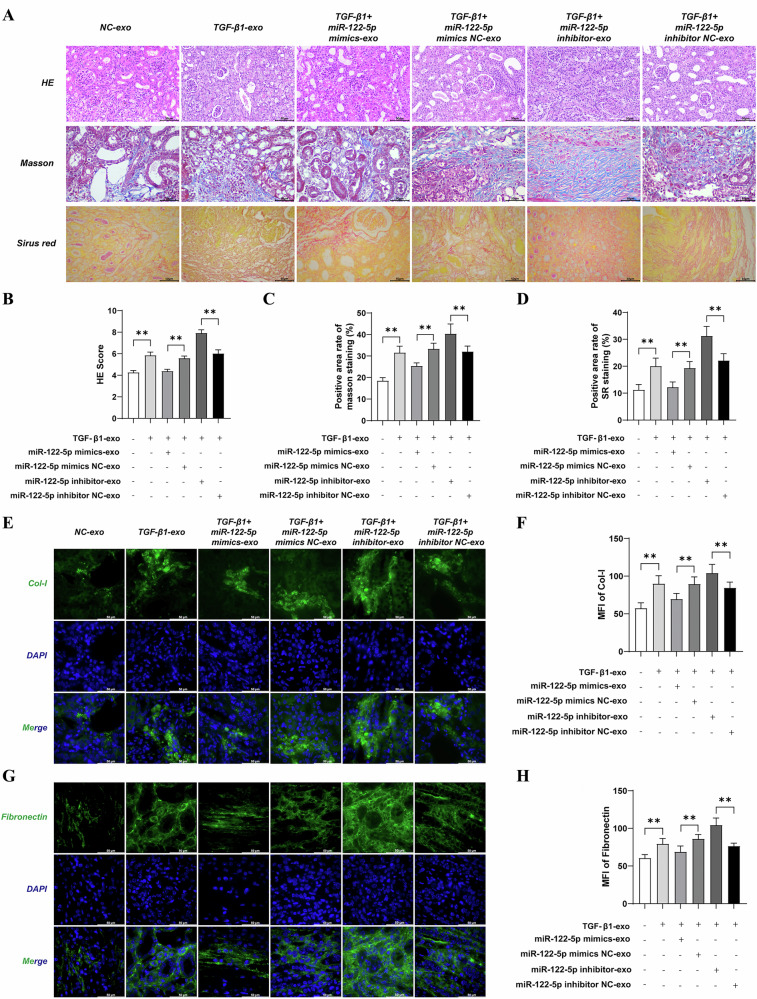
Effects of Overexpressing or Inhibiting miR-122-5p in Exosomes on Renal Fibrosis and Collagen Deposition in UUO Mice. **A** Pathological data yielded by HE staining, Masson staining and Sirius red staining (Bar = 50 μm); **B** HE staining statistics; **C** Masson staining statistics; **D** Sirius red staining statistics; **E**, **F** The expression levels of Col-I in kidney tissue were determined by immunofluorescence (Bar = 50 μm); **G**, **H** The expression levels of Fibronectin in kidney tissue were determined by immunofluorescence (Bar = 50 μm). Results are shown as mean ± SD for six individual experiments. ^**^P < 0.01.

Next, we used HE, Masson’s, and SR staining to evaluate the role of exosomes in the regulation of renal fibrosis. When compared with the NC-exo group, the TGF-β1-exo group exhibited a significantly higher level of interstitial damage, collagen deposition, and fibrosis in the kidney tissue (*P* < 0.01). Compared with the TGF-β1-exo group, there were no significant changes in interstitial damage, collagen deposition, and fibrosis in kidney tissue in the TGF-β1 + miR-122-5p mimic NC-exo and TGF-β1 + miR-122-5p inhibitor NC-exo groups (*P* > 0.01). However, compared with the TGF-β1 + miR-122-5p mimic NC-exo group, the TGF-β1 + miR-122-5p mimic-exo group showed a significant reduction in interstitial damage, collagen deposition, and fibrosis in the kidney tissue (*P* < 0.01). Conversely, compared with the TGF-β1 + miR-122-5p inhibitor NC-exo group, the TGF-β1 + miR-122-5p inhibitor-exo group showed a significant increase in interstitial damage, collagen deposition, and fibrosis in kidney tissue (*P* < 0.01) (Fig. 4A–D).

Compared with the NC-exo group, immunofluorescent analysis showed that the expression levels of Col-I and Fibronectin in the kidney tissue was significantly increased in the TGF-β1-exo group (*P* < 0.01). Compared with the TGF-β1-exo group, there were no significant changes in the expression of Col-I and Fibronectin in the kidney tissue in the TGF-β1 + miR-122-5p mimic NC-exo and TGF-β1 + miR-122-5p inhibitor NC-exo groups (*P* > 0.01). However, compared with the TGF-β1 + miR-122-5p mimic NC-exo group, the expression levels of Col-I and Fibronectin was significantly reduced in the TGF-β1 + miR-122-5p mimic-exo group in kidney tissue (*P* < 0.01), but was significantly increased in the TGF-β1 + miR-122-5p inhibitor-exo group compared with the TGF-β1 + miR-122-5p inhibitor NC-exo group (*P* < 0.01). Collectively, these results indicated that exosomal miR-122-5p has the ability to regulate RIF at the animal level (Fig. 4E–H).

### HIF-1α was identified as a potential target of miR-122-5p

**Fig. 5:**
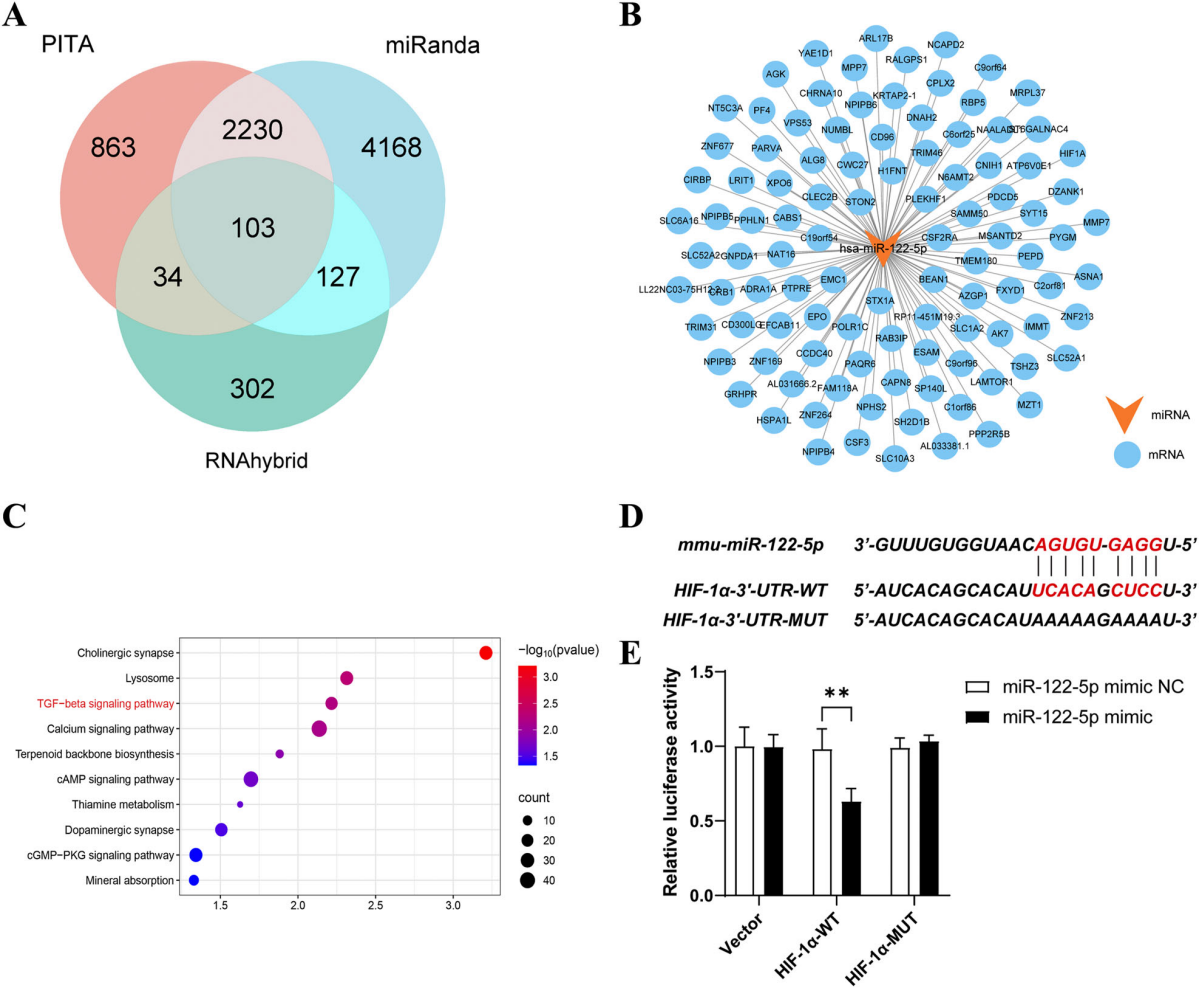
HIF-1α was identified as a potential target of miR-122-5p. **A** The Venn diagram shows the results of three different prediction tools (PITA, miRanda, and RNAhybrid) that were used to predict the target genes of miR-122-5p; **B** A miRNA-mRNA target gene network diagram is shown, featuring the relationship between miR-122-5p and its predicted target gene (mRNA); **C** Enrichment analysis was performed on the genes predicted by miR-122-5p; **D** Predicted potential miR-122-5p binding site in the 3′-UTR of HIF-1α mRNA; **E** Luciferase activity in NRK-49F cells transfected with negative control or miR-122-5p mimic together with a reporter vector containing HIF-1α-mut binding sequences. Results are shown as mean ± SD for three individual experiments. **P < 0.01.

Our findings demonstrated that exosomal miR-122-5p has the ability to regulate RIF at both the cellular and animal level. Next, we investigated the potential mechanism by which renal tubular cell-derived exosomal miR-122-5p could regulate the activation of fibroblasts. PITA, miRanda, and RNAhybrid software were then used to predict the targets of miR-122-5p and identify their intersection (Fig. 5A). HIF-1α is a potential target gene for miR-122-5p (Fig. 5B). Functional enrichment analysis further revealed that the miRNA target genes were significantly enriched in multiple signal transduction and metabolism-related pathways, especially the TGF-β signaling pathway (Fig. 5C). These data indicate that these pathways may change under miRNA regulation, thereby affecting the physiological function and response of cells. By screening the relevant literature and performing bioinformatics analysis, we finally selected the *HIF-1α* gene, which is known to be closely related to fibrosis and fibroblast formation, as the target, and carried out subsequent verification. Since the TGF-β1/Smad pathway is a classical pathway for renal fibrosis, we verified the relationship between target miRNAs and the TGF-β1/Smad pathway. Following sequence alignment, we found that miR-122-5p possessed a binding site in the 3′-UTR of HIF-1α. Next, we constructed an HIF-1α wild-type luciferase reporter gene plasmid (HIF-1α-WT) and a corresponding luciferase reporter gene plasmid (HIF-1α-mut) containing mutant binding sites. Subsequently, binding between HIF-1α and miR-122-5p at identified binding sites was verified by luciferase reporter gene experiments (Fig. 5D, E).

### Tubule cell-derived exosomal miR-122-5p regulated fibroblasts via HIF-1α through the TGF-β1/Smad in vitro

**Fig. 6:**
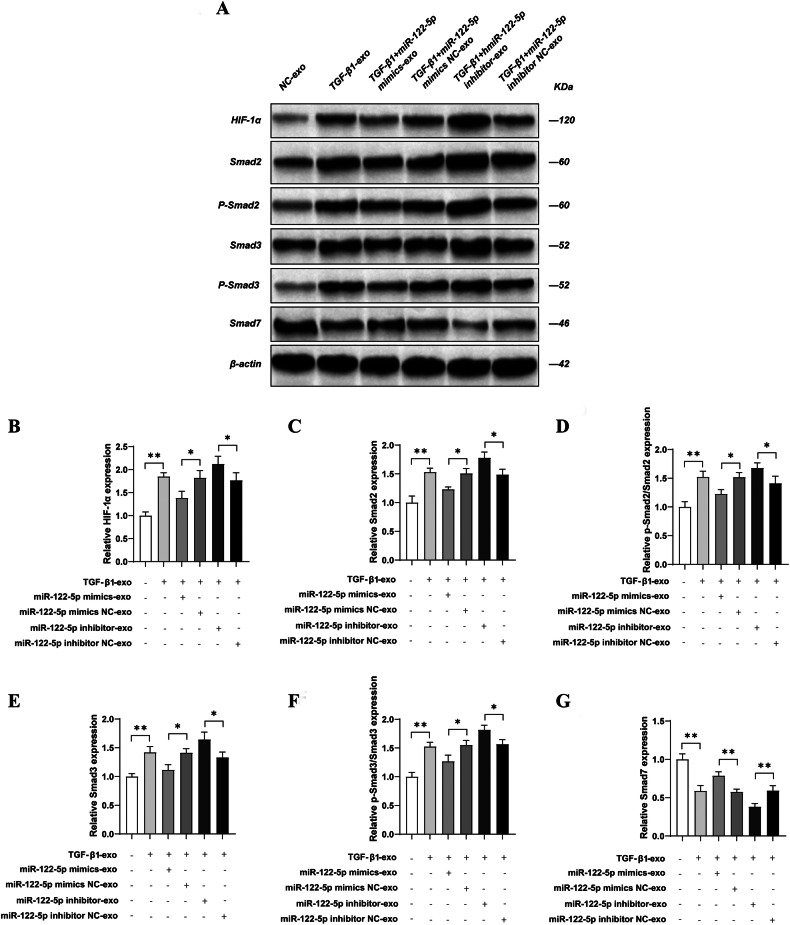
Tubule cell-derived exosomal miR-122-5p regulates fibroblasts via HIF-1α through the TGF-β1/Smad in vitro. **A** The expression of HIF-1α, Smad2, Phospho-SMAD2 (Ser465/Ser467), Smad3, Phospho-SMAD3 (Ser423/425), and Smad7 in NRK-49F cells were detected by western blotting; **B** Relative expression levels of HIF-1α; **C** Relative expression levels of Smad2; **D** Relative expression levels of Phospho-SMAD2 (Ser465/Ser467); **E** Relative expression levels of Smad3; **F** Relative expression levels of Phospho-SMAD3 (Ser423/425); **G** Relative expression levels of Smad7. Results are shown as mean ± SD for three individual experiments. **P < 0.01.

Next, we validated the target and pathway of miR-122-5p at the cellular level. Compared with the NC-exo group, western blotting showed that the expression levels of HIF-1α, Smad2, p-Smad2, Smad3, and p-Smad3 were significantly increased (*P* < 0.01), while the expression level of Smad7 was significantly reduced in NRK-49F cells in the TGF-β1-exo group (*P* < 0.01). Compared with the TGF-β1-exo group, there was no significant change in the expression levels of HIF-1α, Smad2, p-Smad2, Smad3, p-Smad3, E-cadherin, and Smad7 in NRK-49F cells in the TGF-β1 + miR-122-5p mimic NC-exo and TGF-β1 + miR-122-5p inhibitor NC-exo groups (*P* > 0.01). However, compared with the TGF-β1 + miR-122-5p mimic NC-exo group, the expression levels of HIF-1α, Smad2, p-Smad2, Smad3, and p-Smad3 were significantly reduced (*P* < 0.01), while the expression level of E-cadherin was significantly increased in the TGF-β1 + miR-122-5p mimic group in NRK-49F cells (*P* < 0.01). Conversely, compared with the TGF-β1 + miR-122-5p inhibitor NC-exo group, the expression levels of HIF-1α, Smad2, p-Smad2, Smad3, and p-Smad3 expression were significantly increased (*P* < 0.01), while the expression level of Smad7 was significantly reduced in the TGF-β1 + miR-122-5p inhibitor-exo group in NRK-49F cells (*P* < 0.01) (Fig. 6A–G).

### Tubule cell-derived exosomal miR-122-5p regulated fibroblasts via HIF-1α through the TGF-β1/Smad in vivo

**Fig. 7:**
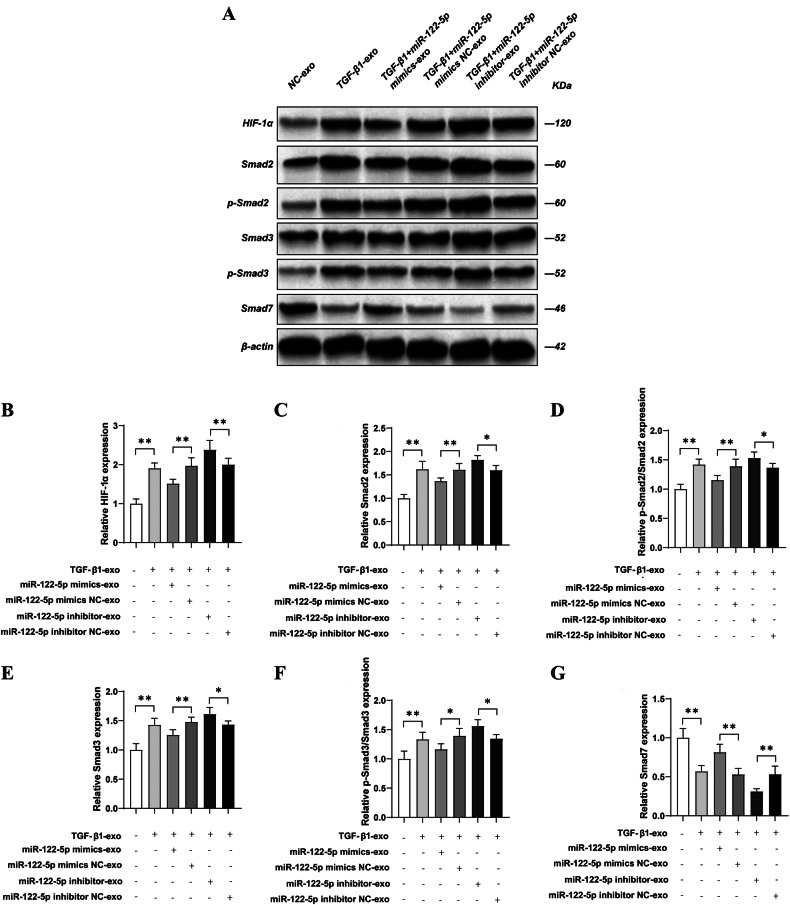
Tubule cell-derived exosomal miR-122-5p regulates fibroblasts via HIF-1α through the TGF-β1/Smad in vivo. **A** The expression levels of HIF-1α, Smad2, Phospho-SMAD2 (Ser465/Ser467), Smad3, Phospho-SMAD3 (Ser423/425), and Smad7 in kidney tissue were detected by western blotting. **B** Relative expression levels of HIF-1α; **C** Relative expression levels of Smad2; **D** Relative expression levels of Phospho-SMAD2 (Ser465/Ser467); **E** Relative expression levels of Smad3; **F** Relative expression levels of Phospho-SMAD3 (Ser423/425); **G** Relative expression levels of Smad7. Results are shown as mean ± SD for six individual experiments. *P < 0.05, **P < 0.01.

Next, we validated the target and pathway of miR-122-5p at the animal level. Compared with the NC-exo group, western blotting revealed that the expression levels of HIF-1α, Smad2, p-Smad2, Smad3, and p-Smad3 was significantly increased (*P* < 0.01), while the expression level of Smad7 was significantly reduced in kidney tissue from the TGF-β1-exo group (*P* < 0.01). Compared with the TGF-β1-exo group, there was no significant change in the expression levels of HIF-1α, Smad2, p-Smad2, Smad3, p-Smad3, E-cadherin, and Smad7 in kidney tissue from the TGF-β1 + miR-122-5p mimic NC-exo and TGF-β1 + miR-122-5p inhibitor NC-exo groups (*P* < 0.01). However, compared with the TGF-β1 + miR-122-5p mimic NC-exo group, the expression levels of HIF-1α, Smad2, p-Smad2, Smad3, and p-Smad3 was significantly reduced (*P* < 0.01), while the expression levels of Smad7 were significantly increased in the TGF-β1 + miR-122-5p mimic-exo group in kidney tissue (*P* < 0.01). Conversely, compared with the TGF-β1 + miR-122-5p inhibitor NC-exo group, the expression levels of HIF-1α, Smad2, p-Smad2, Smad3, and p-Smad3 were significantly increased (*P* < 0.01), and the expression level of Smad7 was significantly reduced in the TGF-β1 + miR-122-5p inhibitor-exo group in kidney tissue (*P* < 0.01) (Fig. 7A–G).

### The overexpression of HIF-1α reversed the ameliorative effect of MiR-122-5p mimic on a TGF-β1 exosome-induced NRK-49F cell model of fibrosis

**Fig. 8:**
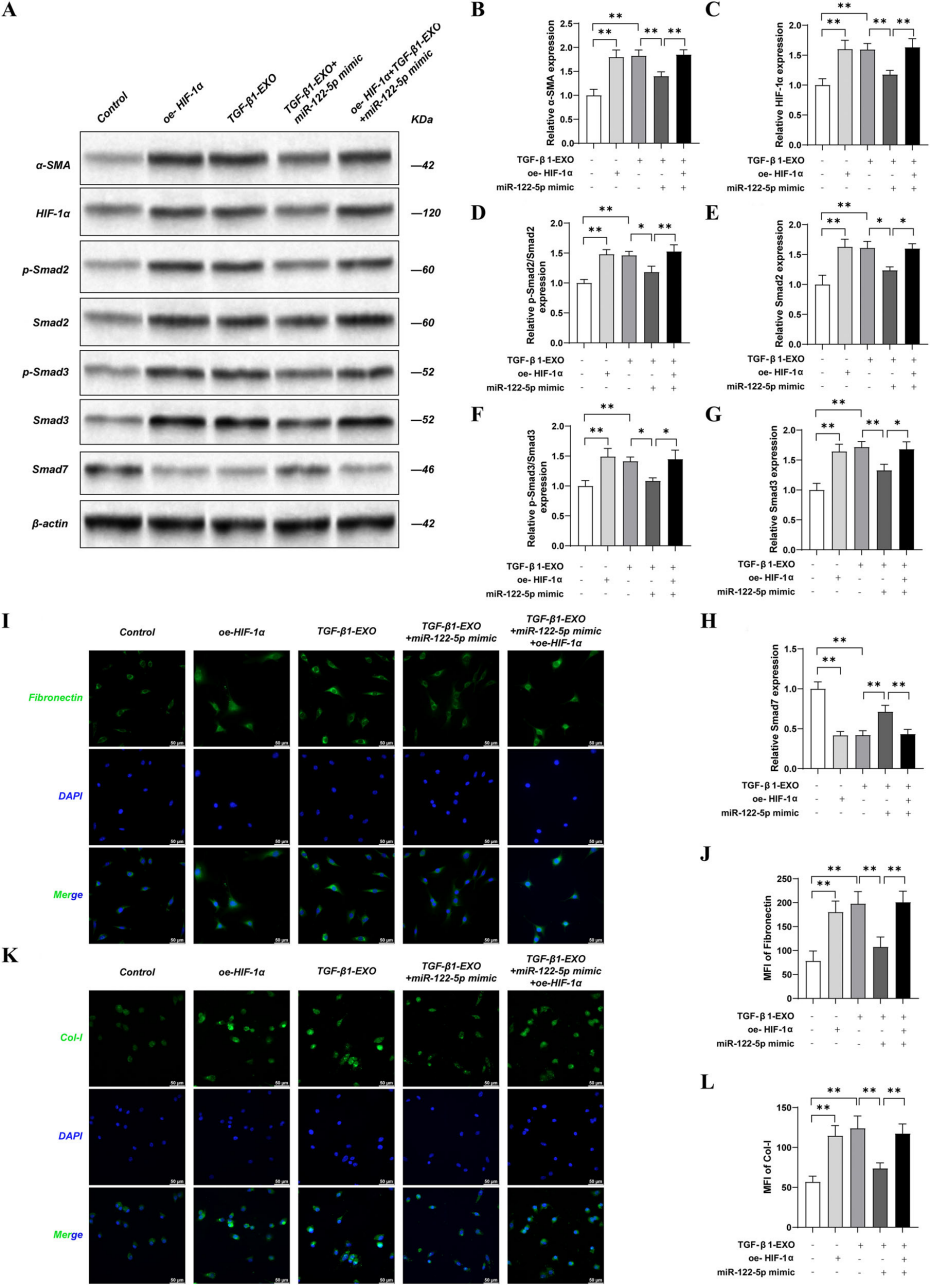
The overexpression of HIF-1α reversed the ameliorative effect of MiR-122-5p mimic on a TGF-β1 exosome-induced NRK-49F cell model of fibrosis. **A** The expression levels of α-SMA, HIF-1α, Phospho-SMAD2 ^(Ser465/Ser467)^, Smad2, Phospho-SMAD3 ^(Ser423/425)^, Smad3, E-cadherin, and Smad7 in NRK-49F cells were detected by western blotting. **B** Relative expression levels ofα-SMA; **C** Relative expression levels of HIF-1α; **D** Relative expression levels of Smad2; **E** Relative expression levels of Phospho-SMAD2 ^(Ser465/Ser467)^; **F** Relative expression levels of Smad3; **G** Relative expression levels of Phospho-SMAD3^(Ser423/425)^; **H** Relative expression levels of Smad7; **I** The expression levels of Fibronectin in NRK-49F cells were detected by immunofluorescence (Bar = 50 μm); **J** Statistics of fibronectin expression levels in NRK-49F cells; **K** The expression levels of Col-I in NRK-49F cells were detected by immunofluorescence (Bar = 50 μm); **L** Statistics of Col-I expression levels in NRK-49F cells. Results are shown as mean ± SD for three individual experiments. ^**^P < 0.01.

To further confirm the effect of miR-122-5p on HIF-1α, we transfected NRK-49F cells with an HIF-1α overexpression plasmid before receiving exosomes delivered by HK-2 cells. Compared with the control group, the oe-HIF-1α group and the TGF-β1-exo group exhibited significantly increased expression levels of α-SMA, HIF-1α, Smad2, p-Smad2, Smad3, and p-Smad3 (*P* < 0.01) and significantly decreased expression levels of E-cadherin and Smad7 in NRK-49F cells (*P* < 0.01). Compared with the TGF-β1-exo group, the TGF-β1-exo + miR-122-5p mimic group showed significantly reduced expression levels of α-SMA, HIF-1α, Smad2, p-Smad2, Smad3, and p-Smad3 (*P* < 0.01) and significantly increased expression levels of E-cadherin and Smad7 in NRK-49F cells (*P* < 0.01). In comparison with the TGF-β1-exo + miR-122-5p mimic group, the oe-HIF-1α + TGF-β1-exo + miR-122-5p mimic group exhibited significantly increased expression levels of α-SMA, HIF-1α, Smad2, p-Smad2, Smad3, and p-Smad3 (*P* < 0.01) and significantly reduced expression levels of E-cadherin and Smad7 in NRK-49F cells (*P* < 0.01) (Fig. 8A–H).

Compared with the Control group, immunofluorescent analysis revealed that the expression levels of Col-I and Fibronectin were significantly increased in NRK-49F cells from the oe-HIF-1α group and the TGF-β1-exo group (*P* < 0.01). Compared with the TGF-β1-exo group, the expression levels of Col-I and Fibronectin were significantly reduced in the TGF-β1-exo + miR-122-5p mimic group in NRK-49F cells (*P* < 0.01). In comparison with the TGF-β1-exo + miR-122-5p mimic group, the oe-HIF-1α + TGF-β1-exo + miR-122-5p mimic group showed significantly increased expression levels of Col-I and Fibronectin in NRK-49F cells (*P* < 0.01). Collectively, these results indicate that exosomal miR-122-5p from tubular cells can regulate fibroblast activation by targeting HIF-1α via the TGF-β1/Smad pathway (Fig. 8I–L).

## Discussion

**Fig. 9:**
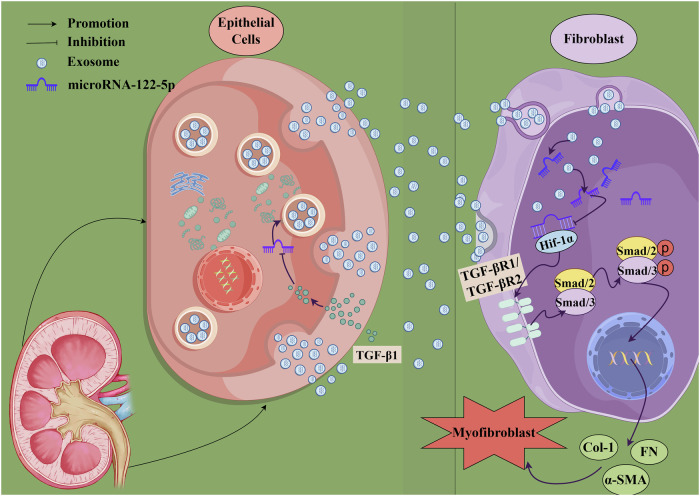
Mechanistic diagram showing the effect of exosomes secreted by renal tubular epithelial cells on fibroblasts after fibrotic action.

In this study, we investigated the role and mechanism of miR-122-5p in CKD RIF. Analysis revealed that the level of miR-122-5p was significantly downregulated in exosomes extracted from TGF-β1-stimulated HK-2 cells and renal tissues obtained from UUO mice. We investigated the effect of exosomes derived from HK-2 cells on NRK-49F cell fibrosis at the cellular level and explored the potential mechanism involved. We extracted exosomes secreted by HK-2 cells and administered them via tail vein injection to UUO mice to further validate the impact of HK-2 cell-derived exosomes on renal fibrosis at the whole animal level. Our experiments demonstrated that exosome-mediated miR-122-5p exerted an anti-fibrotic effect in both in vitro and in vivo models of RIF. Furthermore, we further demonstrated that the miR-122-5p, which was enriched in exosomes from renal tubular epithelial cells, is one of the key molecules regulating fibrotic renal fibroblasts *via* the miR-122-5p/HIF-1α/Smad pathway. Our findings provide new insights into the role of exosomes as epithelial-mesenchymal communication messengers in improving the pathological mechanism of RIF (Fig. 9).

Previous studies have confirmed that exosomes, as important messengers of intercellular communication, play a crucial role in the pathological process of renal fibrosis. During fibrosis, stimulated resident kidney cells, such as epithelial cells, endothelial cells, immune cells, and fibroblasts, engage in complex cellular interactions *via* exosomes to regulate fibrosis [22]. EMT is a multifaceted biological process that mainly manifests as the transformation of epithelial cells to mesenchymal cells and acquires unique phenotypic features. This process is closely related to a variety of physiological and pathological states. Exosomes released from puromycin-treated podocytes have been shown to contain miR-149, -424, -542, -582, and -874, which can promote EMT in renal tubular epithelial cells, thereby contributing to the progression of renal fibrosis [23, 24]. Previous studies showed that miR-21 from multiple myeloma (MM) cell-derived exosomes can promote renal EMT by targeting the TGF-β/SMAD7 signaling pathway [25]. Experimental evidence suggests that exosome-delivered miR-26a binds directly to the 3′-UTR of connective tissue growth factor, reducing its expression level and inhibiting the activation of SMAD3, thereby alleviating aldosterone-induced renal TIF [26]. Therefore, the role of exosomes in RIF warrants significant research attention.

In this study, we first performed high-throughput sequencing analysis, which revealed that multiple miRNAs were differentially expressed in exosomes derived from TGF-β1-induced renal tubular epithelial cells compared to controls. Pathway enrichment analysis of these differentially expressed miRNAs was conducted using bioinformatics approaches. Based on literature review and a rigorous filtering strategy, miR-122-5p was identified as being strongly associated with renal fibrosis and selected for further investigation. Subsequently, we explored the potential regulatory role of exosomal miR-122-5p in renal interstitial fibrosis through both in vitro and in vivo experiments. It is worth noting that we are not the first to investigate the role of miR-122-5p in renal fibrosis, previous research has already demonstrated that miR-122-5p plays an important role in regulating the progression of kidney diseases. In a model of uric acid nephropathy, miR-122-5p was shown to reduce the expression levels of IL-1b and IL-18, thus contributing to the regulation of apoptosis and inflammasome activation [27]. Previous studies, involving a mouse model of renal fibrosis induced by UUO, showed that a miR-122-5p mimic enhanced renal fibrosis by upregulating TGFBR2 [28]. In a model of renal cancer, the overexpression of miR-122-5p was shown to promote renal cancer cell viability, proliferation, migration, glycolysis, and autophagy by negatively regulating PKM2 [29]. In cell and animal models of renal fibrosis, miR-122-5p has been shown to mitigate fibrosis and reduce apoptosis by targeting PKM [30]. Furthermore, miR-122-5p was shown to target transforming growth factor-beta receptor II to negatively regulate the TGF-β1/Smad signaling pathway in skeletal muscle fibrosis, thereby alleviating fibrosis [31]. In a rabbit model of partial UUO, the anti-inflammatory and anti-fibrotic effects of miR-122-5p were suppressed, thus suggesting that miR-122-5p exerts anti-inflammatory and anti-fibrotic effects by inhibiting the expression of SOX2 [32]. Furthermore, miR-122-5p has been shown to negatively regulate the expression of HIF-1α in experimental mice, thereby inhibiting TGF-β1-induced differentiation and collagen synthesis in cardiac fibroblasts and suppressing myocardial fibrosis. Nevertheless, our study remains highly significant [33]. First, our results suggest that miR-122-5p may play different roles in fibrosis across different organs, thus necessitating further experimental validation of the mechanisms involved. Second, previous studies have mostly focused on miR-122-5p levels within kidney tissue; only limited research has addressed miR-122-5p in exosomes. Previous experiments did not explore the intervention of exosomal miR-122-5p or the mechanisms underlying exosome secretion. Most importantly, our study focuses on the role of exosome-mediated epithelial cell-fibroblast communication, a crucial step in the progression of RIF. Thus, our research holds substantial novelty and significance.

The kidney’s vital anatomical structure and physiological functionality make it particularly sensitive to hypoxic pathophysiological processes. Hypoxia accompanies various stress stimuli and disease progression in the kidney. During renal fibrosis, the hypoxia-inducible factor (HIF) family is mobilized and activated under hypoxic conditions, regulating multiple signaling molecules to respond promptly to hypoxia and playing a crucial role in response and regulation [34]. In the kidney, HIF-1α is expressed in most cell types, including proximal tubules, distal tubules, connecting tubules, and collecting ducts, with a wide array of functions, including regulating inflammation, fibrosis, apoptosis, and hypoxia-induced glycolysis in kidney diseases [35, 36]. Previous studies have shown that in models of renal fibrosis, the inhibition of HIF-1α expression can improve metabolic remodeling, effectively alleviating renal fibrosis [37]. Studies have also shown that under hypoxic conditions, HIF-1α participates in the onset and progression of renal fibrosis by regulating cellular adaptive capacity [38]. The genetic knockout of HIF-1α in renal epithelial cells has been shown to reduce the deposition of collagen, a fibrosis-related marker protein. However, one study reported that while HIF-1α affects renal endothelial cells in vitro, it does not influence inflammation and fibrosis progression after UUO. Thus, the role of HIF-1α in renal fibrosis may vary and requires further experimental confirmation. Our study demonstrated that the expression of HIF-1α expression increases during fibrosis, along with the increased expression of fibrosis marker proteins, such as α-SMA, Col-I, and Fibronectin. In addition, we overexpressed HIF-1α and further confirmed the regulatory relationship between miR-122-5p and HIF-1α in vitro. miR-122-5p negatively regulates HIF-1α, inhibiting its expression and thereby mitigating the extent of renal fibrosis. In this study, we employed bioinformatics analysis combined with stringent filtering criteria and both in vitro and in vivo experiments to identify and validate HIF-1α as a direct target of miR-122-5p. Furthermore, we confirmed the expression changes of key proteins in the TGF-β1/Smad signaling pathway, providing a relatively comprehensive mechanistic insight into the role of renal tubular cell-derived exosomal miR-122-5p in renal interstitial fibrosis.

Nevertheless, this study has certain limitations. As a complex intercellular communication vehicle, exosomes carry a wide range of bioactive contents and may exert pleiotropic effects. Although the source of the exosomes used in our study is well-defined, we did not experimentally validate other potential target genes or signaling pathways that may also be regulated by miR-122-5p. Moreover, we cannot completely exclude the possibility that other components within exosomes might also contribute to the observed anti-fibrotic effects. These aspects will be further explored in future studies. This study focuses on in vivo and in vitro experiments, and is not related to clinical diseases. In the future, we can include kidney tissue, urine, blood and other samples from patients with nephropathy for further exploration and verification.

## Conclusion

Our analyses demonstrated that exosomes derived from renal tubular cells play an important role in alleviating RIF. We found that in models of fibrosis, the production of miR-122-5p in exosomes was reduced. Exosomal miR-122-5p from renal tubular cells targeted HIF-1α, attenuated fibroblast activation, and influenced the TGF-β1/Smad pathway. These findings identify the mechanism of exosome-mediated cell-to-cell communication, providing new insights and approaches to slow or mitigate the progression of renal fibrosis.

## Materials and methods

### Animal model

C57BL/6J mice (20–25 g) were provided by Jiangsu Huachuang Xinnuo Pharmaceutical Science and Technology Co (JiangSu, China). The Laboratory Animal Production License number was SYXK (Su) 2021-0007 and the Laboratory Use License number was SCXK (Su) 2021-0007. The mice were housed in a specific pathogen-free (SPF) facility with a 12 hour (h) light/dark cycle and ambient temperature of 22 °C. Food and water provided ad libitum. A mouse model of unilateral ureteral obstructed (UUO) was established by unilateral ureteral ligation in accordance with methods described previously [39]. Once the model had been established, the mice were randomized into a sham group and a UUO-14d group. Kidneys from the two groups were removed 14 days after modeling, and kidney tissues were collected for various analyses. All animal procedures were approved by the Animal Welfare and Ethics Committee of Hebei University (Approval No. IACUC2021003SM). Prior to surgical procedures, including UUO induction, mice were anesthetized via intraperitoneal injection of pentobarbital sodium (50 mg/kg). Humane endpoints were established prior to experimentation. Mice were monitored daily, and if any exhibited >20% body weight loss, persistent anorexia, severe infection, or respiratory distress, humane euthanasia was performed under deep anesthesia via cervical dislocation. No unexpected adverse events occurred during the study. All procedures complied with the principles of minimizing animal suffering and reducing the number of animals used. To investigate the specific role of exosomes, we quantified the exosomes produced by HK-2 cells treated with or without TGF-β1 and then injected these exosomes intravenously at UUO-14d (100 µg per mouse per time point). miR-122-5p was previously transfected into HK-2 cells.

The experimental design was as follows: There are six subgroups: NC group (HK-2 cells treated without TGF-β1), TGF-β1group (HK-2 cells treated with TGF-β1), TGF-β1+ miR-122-5p mimics group (HK-2 cells treated with TGF-β1 and transfected with miR-122-5p mimics), TGF-β1+ miR-122-5p mimic NC group (HK-2 cells treated with TGF-β1 and transfected with miR-122-5p mimics NC), TGF-β1+ miR-122-5p inhibitor group (HK-2 cells treated with TGF-β1 and transfected with miR-122-5p inhibitor), TGF-β1+ miR-122-5p inhibitor NC group (HK-2 cells treated with TGF-β1 and transfected with miR-122-5p inhibitor NC). Exosomes from each of the six groups were then finally injected intravenously into the sham group (*N* = 6) and UUO-14d group (*N* = 6).

### Cell line and cell culture

Human Kidney-2 (HK-2) cells and rat fibroblast (NRK-49F) cells (ATCC, Rockville, MD, USA) were cultured in 10% fetal bovine serum (FBS, Hyclone, Logan, UT, USA) and Dulbecco’s modified Eagle medium (DMEM) (D0819) at 37 °C with 5% CO_2_ in a humidified environment. HK-2 and NRK-49F cells were both incubated in exosome-free medium for 24 h as negative controls (NCs). In the experimental group, HK-2 cells were treated with 10 ng/mL of TGF-β1 for 24 h by incubation in exosome-free medium and transfected with miR-122-5p mimics, a miR-122-5p inhibitor, or their corresponding NCs. NRK-49F cells were pretreated with 10 ng/mL of TGF-β1 for 2 h and treated with exosomes that had been isolated from HK-2 cells.

### Exosome isolation

Exosomes were purified from conditioned medium using the Ome-01 kit (OMIGET, Beijing, China). Cell culture supernatant was collected and centrifuged at 3000 × *g* for 5 min at 4 °C. The supernatant was transferred to a new tube, and 0.6 mL of vortexed magnetic beads from the kit was added to a 15 mL centrifuge tube, followed by centrifugation at 3000 × *g* for 2 min at 4 °C. Discarding the supernatant, the beads were resuspended and vortexed in 10 mL pre-cooled PBS, and centrifuged at 3000 × *g* for 5 min at 4 °C. A 20 mL purification system was prepared which 15.6 mL sample (topped up with ddH_2_O), 3 mL Buffer EXP, 1 mL Buffer EXN, 0.2 mL Buffer EXT, and 0.2 mL Magnetic Beads. This purification system was mixed by rotation at 4 °C for 60 min, then centrifuged at 3000 × *g* for 5 min at 4 °C. The supernatant was discarded, and the beads were resuspended in 0.3 mL of Buffer EXE and vortexed, centrifuged at 7000 × *g* for 2 min at 4 °C. The purified exosome solution was transferred to a new EP tube after placing on a magnetic rack. Needle filters A and B were rinsed with PBS, and the exosome solution was filtered through these filters to remove impurities, yielding high-purity exosomes stored at −80 °C.

### Nanometer particle tracking analysis (NTA)

Nanoparticle analysis (NanoFCM, Flow NanoAnalyzer, Xiamen, China) was used to characterize the particle concentration, distribution and mean particle size of the secretions.

### Transmission electron microscopy (TEM)

Transmission electron microscopy (TEM) to 10 (including l secrete body solution to copper net. This was then incubated for 10 min at room temperature, cleaned with sterile distilled water, and dried with blotting paper to remove excess liquid. Then, 10 µL of 2% uranyl acetate dropped onto the copper mesh for negative staining for 1 min. The floating liquid was removed by filter paper and dried under an incandescent lamp for 2 min. The copper mesh was then observed by transmission electron microscopy and imaged with 80kv. Final images were observed with a transmission electron microscope at 80 kV (Hitachi, Japan, H7650 TEM).

### High throughput miRNA sequencing

Purified exosomes from HK-2 cells with or without TGF-β1 (10 ng/mL) stimulation separately underwent extraction of total RNA, including the small RNA fraction, and after extraction of the exosomal RNA, high-throughput sequencing was performed by Yanjiang Biotechnology company (Shanghai, China). Total RNA detection, gene library construction, and HiSeq/MiSeq sequencing were carried out according to the manufacturer’s instructions.

### Bioinformatics analysis

To identify potential target genes of miR-122-5p, three miRNA target prediction tools (PITA, miRanda, and RNAhybrid) were used. PITA considers sequence complementarity and binding site accessibility, miRanda focuses on 7-mer seed pairing and alignment scores, and RNAhybrid identifies low-energy binding sites in 3′UTRs. Default thresholds were applied, and only overlapping predictions from all three tools were analyzed to ensure reliability and minimize false positives. To identify enriched functional categories and pathways, the clusterProfiler R package (version 3.1.8)was used for GO and Kyoto Encyclopedia of Genes and Genomes (KEGG) enrichment analyses. GO analysis classified genes into three domains: biological process (BP), molecular function, and cellular component, using the enrichGO function and species-specific annotation databases. KEGG pathway enrichment was performed using the enrich KEGG function, with gene identifiers converted to KEGG-compatible formats. For both analyses, enrichment was determined using a hypergeometric test, and significant terms or pathways were identified based on an *p* value threshold (<0.05).

### Fluorescence labeling of exosomes

HK-2 cells were first labeled with PKH-67 (a form of fluorescent lipophilic cationic indocarbocyanine dye) and washed three times with PBS. PKH-67-labeled exosomes extracted from the conditioned media of HK-2 cells were then incubated with NRK-49F cells for 24 h or injected into mice via the tail vein. Subsequently, exosome distribution was determined by immunofluorescence. Then, we added 4% paraformaldehyde, fixed for 10 min, and then rinsed three times with PBS buffer. Cells were then incubated with drops of DAPI (C0060) (Solarbio, BeiJing, China) staining solution for 3 min at room temperature, rinsed with PBS buffer, and aspirated to remove excess liquid. Then, the cells were observed under a fluorescence microscope; images were acquired randomly from 400 fields of view.

### Overexpression plasmid construction and transfection

The gene segment encoding HIF-1α, spanning 2480 base pairs, was synthesized and subsequently cloned into the pcDNA3.1 vector. A gene fragment containing HIF-1α was synthesized and cloned into the pcDNA3.1 vector (V012531) (NovoPro, ShangHai, China). Corresponding cleavage sites and guard bases were added to both ends of the target gene fragment; the cleavage sites a were ECoRI (GAATTC) and Not1 (GGCCGC). The overexpression plasmid was confirmed by enzyme digestion, annealing, ligation, PCR analysis and DNA sequencing, plasmid extracts and by transfection into cells. The enzyme digestion system was as follows: 10×CutSmart Buffer 2 (5 µL), Purified plasmid DNA (1 µg/µL, 2 µL), ECORI (10 U/µL, 1 µL), Not 1 (10 U/µL, 1 µL). The annealing and ligation system was as follows: ddH_2_O (9.2 µL), linear vector(100 ng/µL, 1 µL),double-stranded DNA (100 ng/µL, 1 µL), 10taining HIF-1α was synthesized and cloned into the pcDNA3.1 vector (V012531) (NovoPro, ShangHai, China, as follows: Step1 (94 °C, 3 min), Step2 (94 °C, 30 s; 55 °C, 30 s; 72 °C,30 s; Total 22 cycles), Step3 (72 °C, 5 min), Step4 (4 °C).

For transfection, cells were seeded into 24-well plates and cultured overnight until 70% confluent. Subsequently, cells were transfected with the indicated overexpression plasmid (200 ng) using a Lipofectamine 3000 kit (L3000150) (0.75 µL). The indicated overexpression plasmid and the Lipofectamine 3000 were incorporated into 150 µL of OPTI-MEM medium (31985062) (Thermo Fisher Scientific, Pittsburgh, PA, USA), respectively. The two systems were mixed well and added to the 24-well plates at 37 °C with 5% CO_2_ in a humidified environment. The medium was changed to a serum-free medium after 6 h. The upregulation of target genes was subsequently confirmed by RNA extraction and qRT-PCR analysis.

### Histology, immunohistochemical and immunofluorescence staining

Paraffin-embedded mouse kidney sections were prepared by a routine procedure. Subsequently, we performed hematoxylin-eosin staining. The prepared tissue paraffin sections were placed in an electrically heated oven and baked for 3 h. The dried paraffin sections were then subjected to conventional xylene (Sinopharm Group Chemical Reagent Co., LTD, Shanghai, China) dewaxing and attained with hematoxylin (G1120) (Solarbio, Beijing, China) for 2 min and Eosin staining solution for 1 min. The sections were then dried by gradient alcohol dehydration and sealed. Images were acquired from 400 random fields of view.

#### Masson’s trichrome staining

The prepared paraffin sections were placed in an electrically heated oven and baked for 3 h. Dried paraffin sections were then subjected to conventional xylene dewaxing. Sections were then dehydrated with a gradient series of ethanol and mounted in a temperature chamber at 60 °C for 1 h. Sections were then washed in distilled water three times (3 min per wash). Staining was then performed dropwise with azurite blue staining solution for 3 min and washed with water for 15 s. Mayer’s hematoxylin staining solution was applied dropwise for 3 min and washed twice with distilled water for 15 s. The sections were then stained with Lichun red magenta staining solution for 10 min, treated with phosphomolybdic acid solution for 10 min, and then dehydrated in 70% ethanol, 80% ethanol, 90% ethanol, and anhydrous ethanol for 10 s. The sections were then cleared three times with xylene (for 1 min each time) and sealed with neutral gum. Images were then acquired from 400 randomly selected fields of view.

#### Sirius red staining

The prepared tissue paraffin sections were placed in an electrically heated oven and baked for 3 h. Dried paraffin sections were then subjected to conventional xylene dewaxing and sections were stained with SR for 15 min and dried by gradient alcohol dehydration, cleared with xylene, and sealed with neutral gum. Images were then acquired from 400 randomly selected fields of view.

For immunofluorescence analysis, pretreated cells (5 × 10^5^ cells/well) were seeded in confocal dishes and fixed with 4% paraformaldehyde (Biosharp, HeFei, China), blocked with blocking buffer, incubated with primary antibodies Collagen I Antibody (ab270993) (Abcam, Cambridge, UK) (1:2000 dilution) or Fibronectin antibody (ab268020) (Abcam, Cambridge, UK) (1: 50 dilution) at 4 °C overnight, and then incubated with Alexa Fluor-conjugated secondary antibodies (Alexa Fluor 488, 1:200 dilution) for 1 h at room temperature. Nuclei were stained with DAPI. The cells were examined under a laser scanning confocal microscope.

### qRT-PCR analysis

First, For cellular mRNA extraction, cells were seeded into 6-well plates and cultured overnight until 90% confluent. Subsequently, total RNA was extracted from cells in each plate with TRIzol Reagent (9109) (Takara, Japan), 0.5 mL per well, and transferred to a microcentifuge tube. Then, we added 0.1 ml of chloroform (1/5 of the volume of Trizol) to each tube, allowed the reaction to stand for 10 min, and then centrifuged at 12000 rpm 4 °C for 15 min. Then, we took the supernatant and added an equal amount of isopropanol (approximately 200 μL); this mixture was shaken vigorously and allowed to stand for 30 min at −20 °C to precipitate RNA. Then, we centrifuged for 10 min at 12,000 rpm 4 °C, and discarded the supernatant. Next, we centrifuged for 10 min at 12,000 rpm at 4 °C and discarded the supernatant. Then, we added 1 mL of 75% ethanol (DEPC water), centrifuged at 7500 rpm 4 °C for 5 min, discarded the supernatant, and dried for 10 min. Next, 10 μL of DEPC water was used to dissolve the RNA; 2 μL of the sample was then taken to determine the mRNA concentration and purity with a Nanodrop 2000.

Second, cDNA was synthesized with an iScript cDNA Synthesis Kit (Bio-Rad, Hercules, CA, USA). The system for reverse transcription was as follows: 5× iScript reaction mix (4 µL), iScript reverse transcriptase (1 µL), RNA (2 µL), and Nuclease-freewater (13 µL). The reaction system was set up with the reaction at 25 °C for 5 min, 46 °C for 20 min, and 95 °C for 1 min, and the cDNA was stored at −70 °C.

Finally, real-time fluorescent quantitative PCR was performed with a StepOne™Real-Time PCR Kit (D1801). Three complex wells were prepared for each sample by real-time PCR using the SYBR Green PCR SuperMix Kit (Haigene, Harbin, China). The PCR primers were as follows: HIF-1α:5′-AAGCAGCAGGAATTGTAAGTGG-3´ (forward), 5×Golden HS SYBR Green qPCR Mix (4 µL), 50×ROX Reference Dye (0.4 µL), 10 μM Forward primer, 10 μM Reverse primer (0.4 µL), cDNA (2.5 µL), ddH_2_O (up to 20 µL). The PCR primers were as follows: HIF-1α: 5′-AAGCAGCAGGAATTGTAAGTGG-3′ (forward), 5′-GAAAGCGACATAGTAGGGGCA-3′ (reverse); β-actin: 5′-CTGTGTGGATTGGTGGCTCT-3′ (forward), 5′-AGCTCAGTAACAGTCCGCCT-3′ (reverse); The qPCR reaction conditions were as follows: Step1 (95 °C, 15 min), Step2 (95 °C, 5 s; 55 °C, 5 s; 70 °C, 30 s; Total 30-40 cycles), Step3 (4 °C, 5 min), Step4 (4 °C, dissociation analysis).

For miRNA analysis, exosomal miRNAs were isolated with a SeraMir Exosome RNA Purification Kit (Ome-01) (OMIGET, BeJing, China). Then, qRT-PCR was performed using FastStart Universal SYBR Green Master Mix with a miRNA-specific forward primer and a universal reverse primer. The PCR primers were as follows: miR-122-5p: 5′-TGGAGTGTGACAATGGTGTTTG-3′ (forward), 5′-CAGGTCCAGTTTTTTTTTTTTTTTVN-3′(reverse); U6: 5′-CTCGCTTCGGCAGCACA-3′(forward), and 5′-AACGCTTCACGAATTTGCGT-3´. The qPCR reaction system and reaction conditions were the same as those described above.

### Western blotting

The concentration of protein extracted from animal tissues or cells was detected using the bicinchoninic acid method. Proteins were then separated by 10% sodium dodecyl sulfate-polyacrylamide gel electrophoresis (SDS-PAGE) (BL517A) (Biosharp, HeFei, China) and then transferred onto a polyvinylidene difluoride (PDVF) membrane for 1–2 h at 120 V. After blocking with Tris Buffered Saline with Tween-20 (TBST) containing 5% dried skimmed milk for 1 h at room temperature (approximately 25 °C), the membranes were incubated with primary antibodies overnight at 4 °C. The primary antibodies were as follows: TSG-101 (Abcam, ab125011), HSP70 (Abcam, ab181606), CD63 (Santa, Ca. sc-5275), and calnexin (Proteintech, Rosemont, IL, 10427-2-AP). Anti-HIF-1α antibody (14179), Anti-Smad2 antibody (5339), Anti-Phospho-SMAD2 (Ser465/Ser467) antibody (18338), Anti-Smad3 antibody (9523), and Anti-Phospho-SMAD3. (Ser423/425) antibody (9520) were purchased from CST (Cell Signaling Technology, Inc.). Boston, USA); Anti-Smad7 antibody (AF5147) was purchased from Affinity Company (Affinity, ChangZhou, China). Anti-E-cadherin antibody (ab231303), Anti-β-actin antibody (ab6276), Goat Anti-Rabbit IgG H&L (HRP) (ab6721), Rabbit Anti-Mouse IgG H&L (HRP) (ab6728), and Goat Anti-Rabbit IgG H&L (Alexa Fluor® 488) (ab150077) were purchased from Abcam (Abcam, Cambridge, UK). Finally, fluorescence signals emitted by the secondary antibody were quantified by a western blot detection system (Odyssey Infrared Imaging; LI-COR Biosciences) and semiquantitative analysis was conducted to determine protein expression levels. Uncropped blots are shown in Supplementary Material File.

### Kidney function tests

The mice were randomized into a sham group (*N* = 6) and a UUO-14d group (*N* = 6). Kidneys from the two groups were removed 14 days after modeling, and kidney tissues were collected for various analyses. Blood samples were collected from the mice in each group, stored at 4 °C overnight, then centrifuged at 3000 rpm for 15 min to collect serum. Subsequently, the serum levels of creatinine and urea nitrogen were determined by a biochemical instrument (PF-300, Shenzhen Pukang Electronics Co).

### In vivo imaging

C57BL/6 mice were randomized into a sham group and a UUO-14d group (*N* = 6). The mice were fed normally for 14 days and then injected intravenously with 2 × 10^10^ particles/g Dir-labeled TGF-β1 group exosomes from HK-2 cells. The distribution of exosomes was then determined by an in vivo imager (ABLX1, Shanghai Tianneng Company) 12 h after injection.

### Luciferase reporter assay

The HIF-1α-3′UTR including an miR-122-5p mimic binding site was constructed by PCR and subsequently cloned into the pMIRGLO-Vector (E1330) to construct a wild-type HIF-1α luciferase reporter. The wild-type HIF-1α construct and the miR-122-5p mimic were then co-transfected into 293 T cells using Lipofectamine 3000. Lysates were then harvested and luciferase activity was measured by a dual-luciferase assay. We took 20 μL of the lysates from each group and added 100 μL of Dual-Lumitm^TM^Firefly Dual-Lumitm^TM^Firefly (E2940) (Promega, Madison, USA). Fluorokinase Assay Reagent was mixed and added appropriately. This was incubated at room temperature (~25 °C) for 5 min to stabilize the luminescence signal. Chemiluminescence detection was then performed with a Multifunctional Enzyme Labeler (Tanon 5200) that was capable of detecting chemiluminescence.

### Statistical analyses

All experimental data are expressed as means ± standard deviation and were analyzed using SPSS 23.0 software and plotted by GraphPad Prism 9 (Version 9.4.0) and Adobe Illustrator (Version 26.3.1). After a test for variance homogeneity, an independent-samples *t* test was used to assess significant differences between groups, and one-way ANOVA was used for multiple groups. The levels of statistical significance were denoted as. *p < 0.05, **p < 0.01, and ***p < 0.001. A *p* value less than 0.05 was considered statistically significant.

### Ethics statement

The animal portion of our work was approved by the Animal Welfare and Ethical Committee of Hebei University (Approval No. IACUC2021003SM). The study was conducted in accordance with the local legislation and institutional requirements.

## Supplementary information

Figure S1

Figure S2

Figure S3

Supplementary Figures Lengends

all Figures Uncropped blots

## Data Availability

The datasets generated and/or analyzed during the current study are available in the NCBI Gene Expression Omnibus (GEO) repository under accession number GSE305699, and are available from the corresponding author upon reasonable request.
