# Supplementary figures and images for: Exosomal miR-122-5p from tubular cells ameliorates renal interstitial fibrosis by regulating fibroblasts via HIF-1α
Source: Cell Death Discov. 2025 Oct 21;11:474. doi: 10.1038/s41420-025-02739-8 (PMC12540884; doi:10.1038/s41420-025-02739-8)

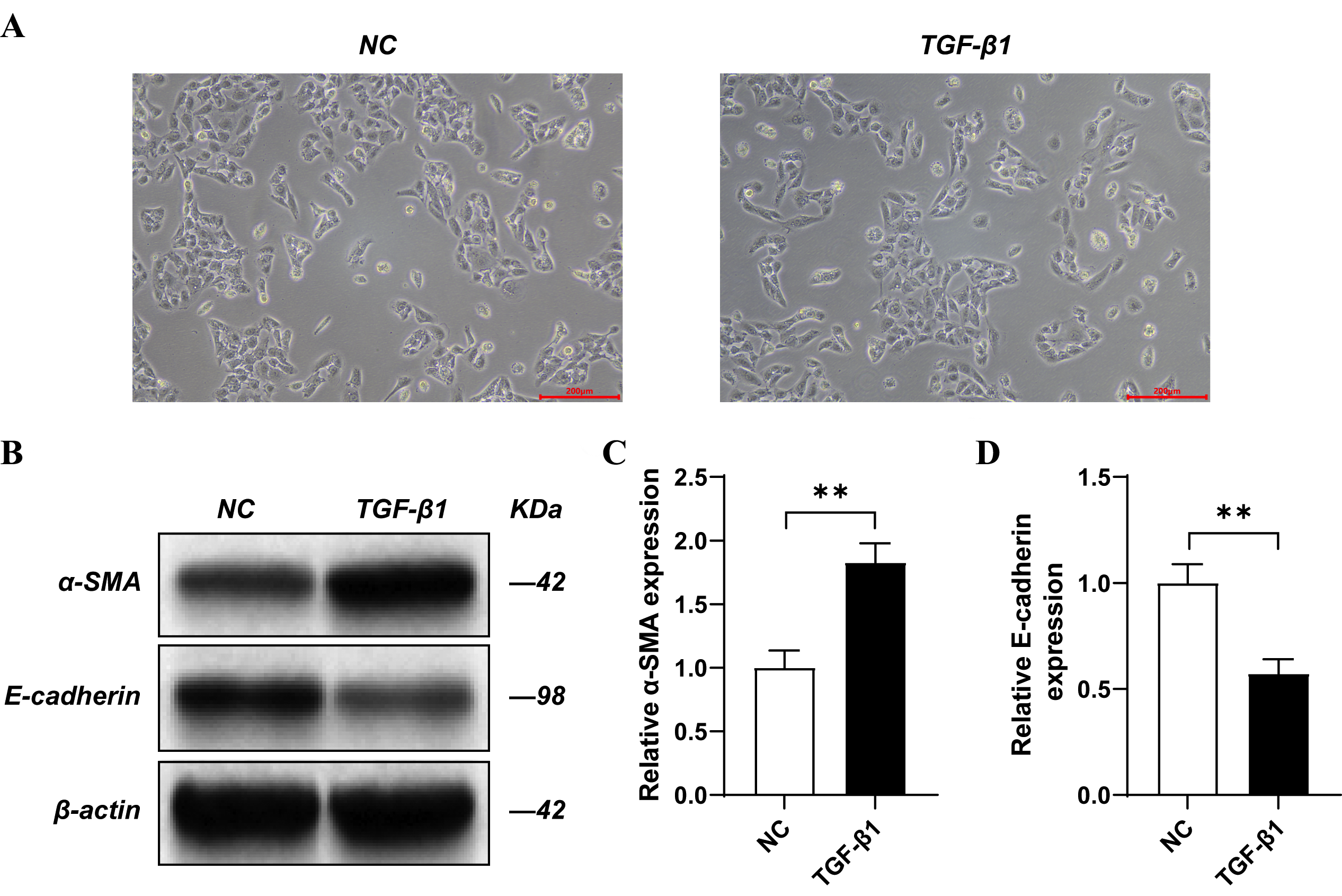

Supplement: Supplementary file 1 — Figure S1 [file 41420_2025_2739_MOESM1_ESM.tif]

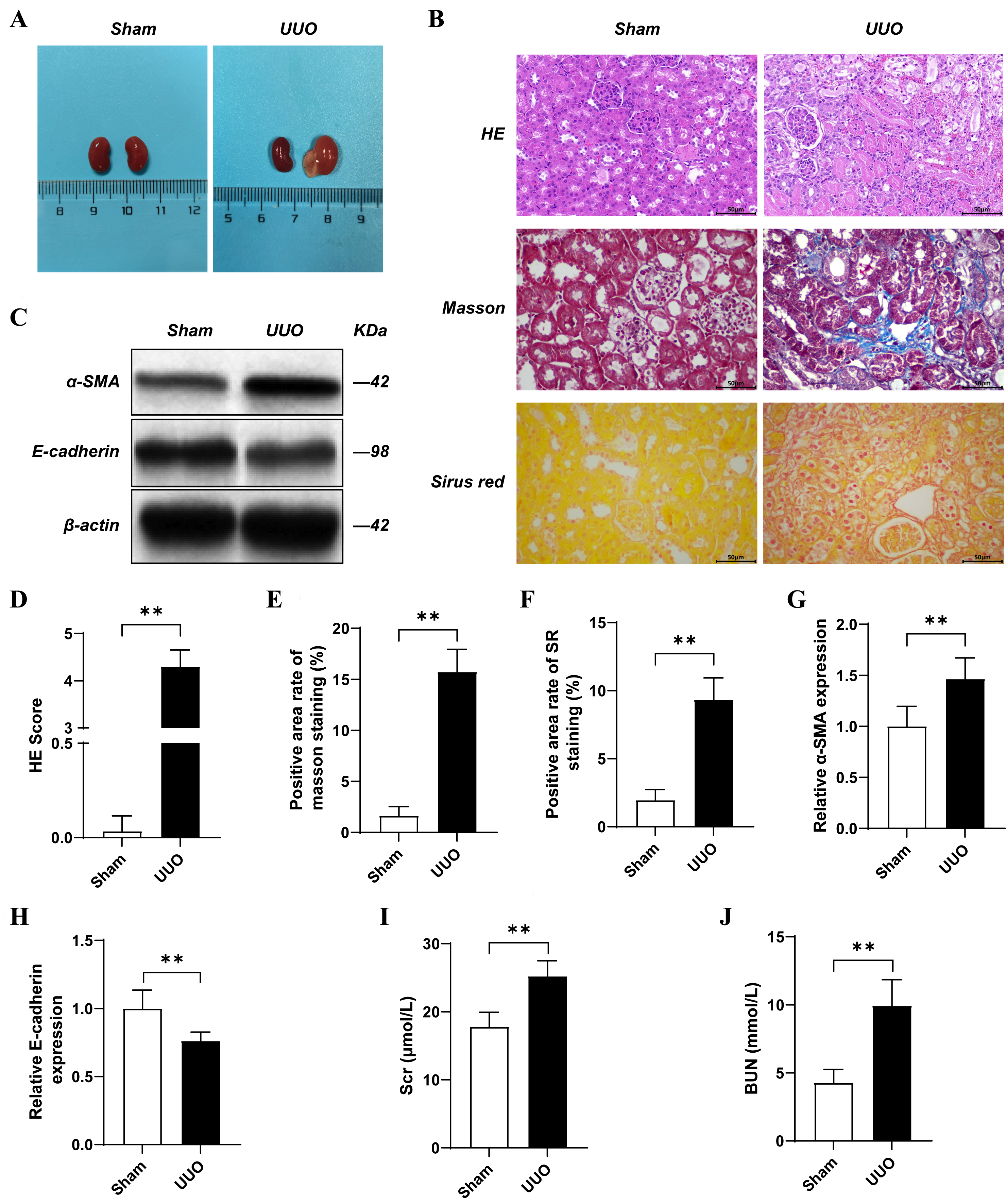

Supplement: Supplementary file 2 — Figure S2 [file 41420_2025_2739_MOESM2_ESM.tif]

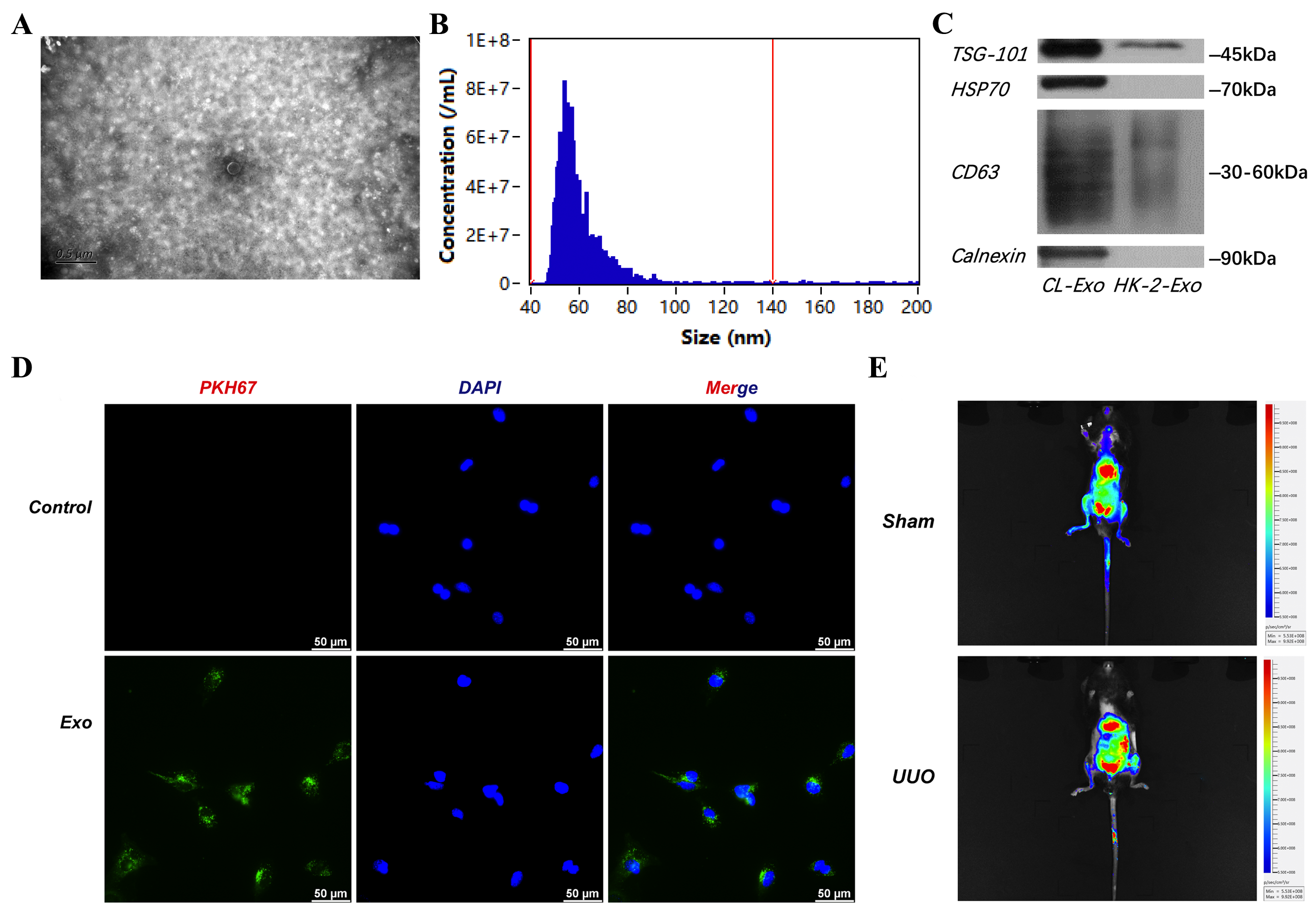

Supplement: Supplementary file 3 — Figure S3 [file 41420_2025_2739_MOESM3_ESM.tif]

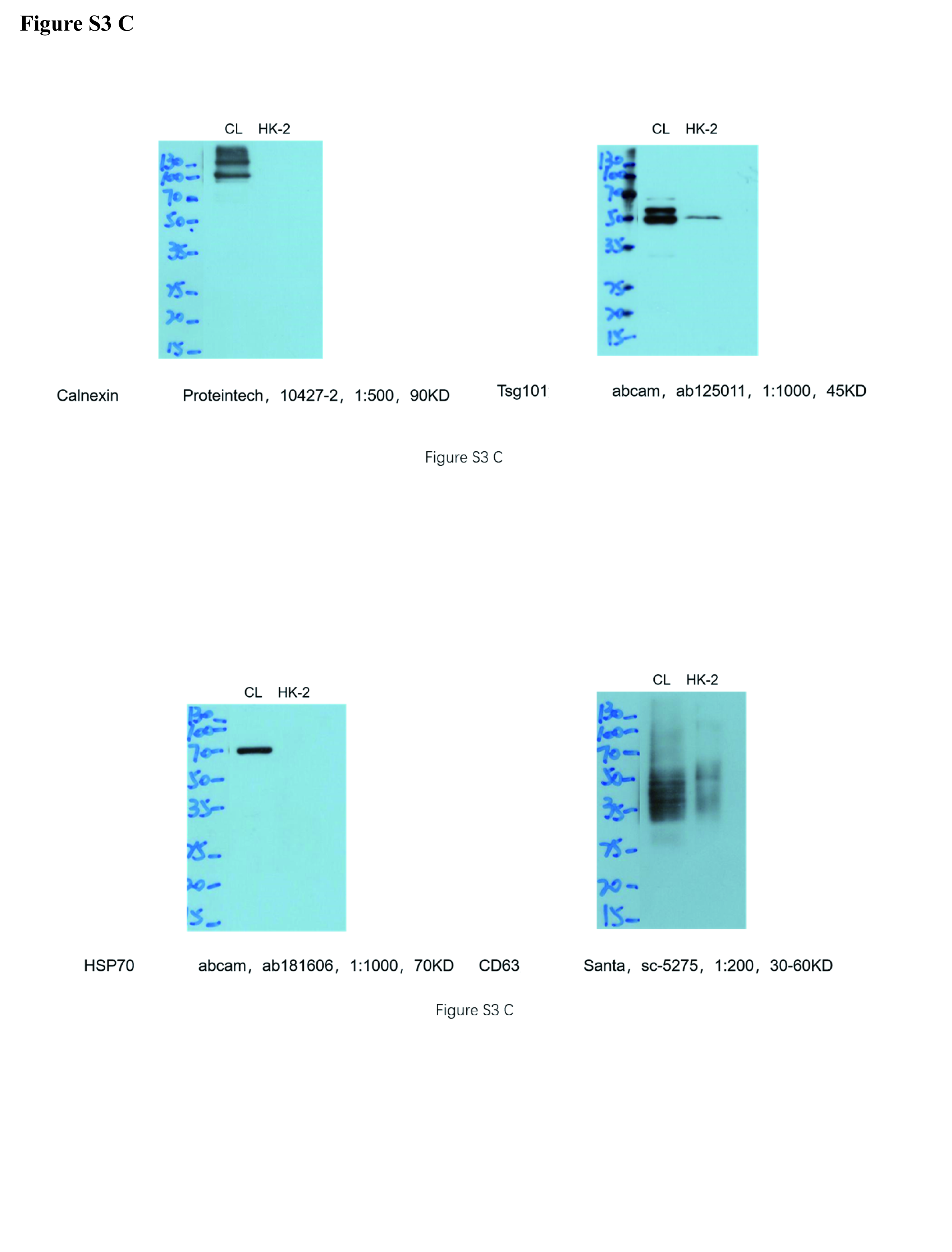

Supplement: Supplementary file 5 — all Figures Uncropped blots [file 41420_2025_2739_MOESM5_ESM.zip › blot-13.tif]

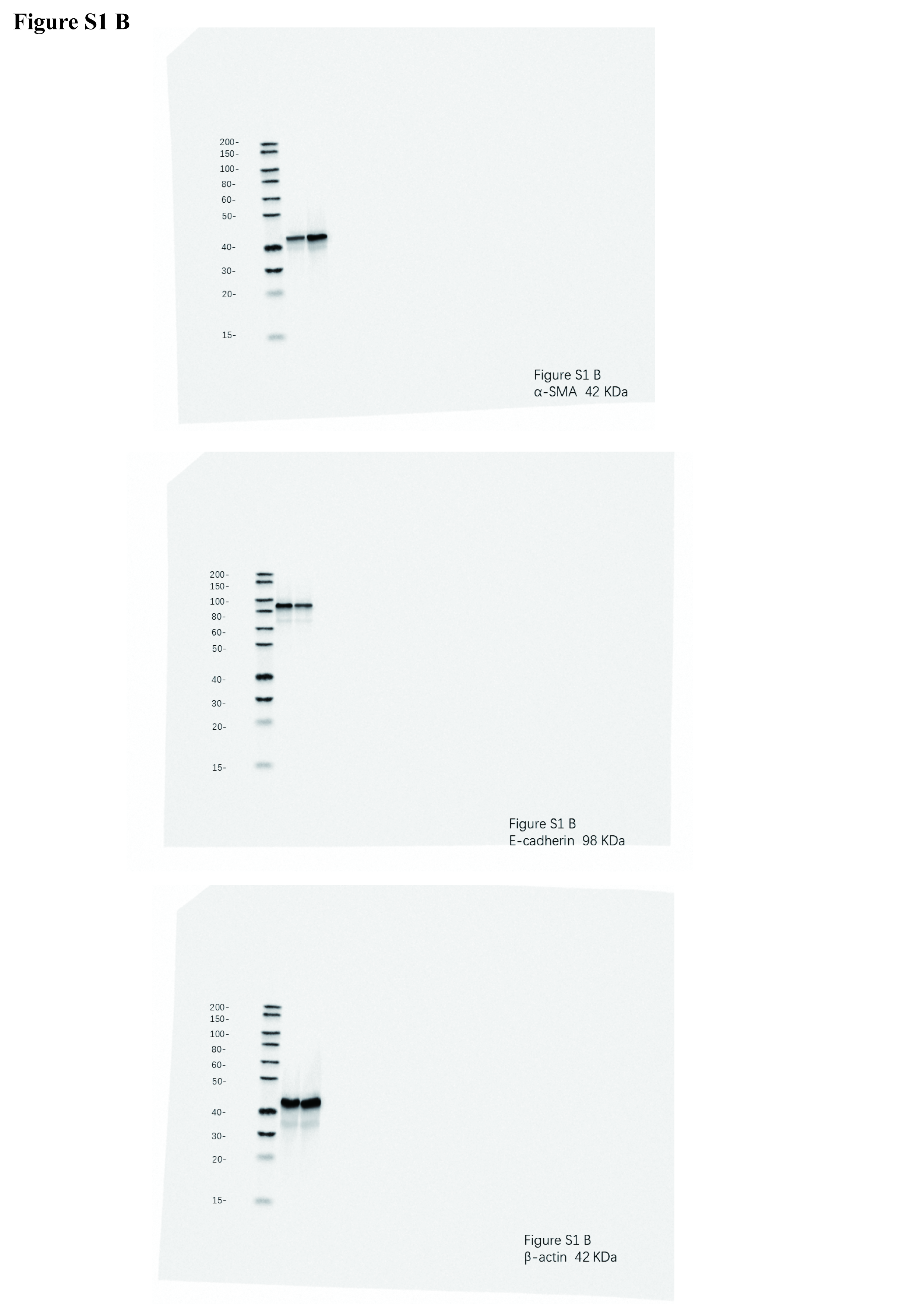

Supplement: Supplementary file 5 — all Figures Uncropped blots [file 41420_2025_2739_MOESM5_ESM.zip › blot- 00.tif]

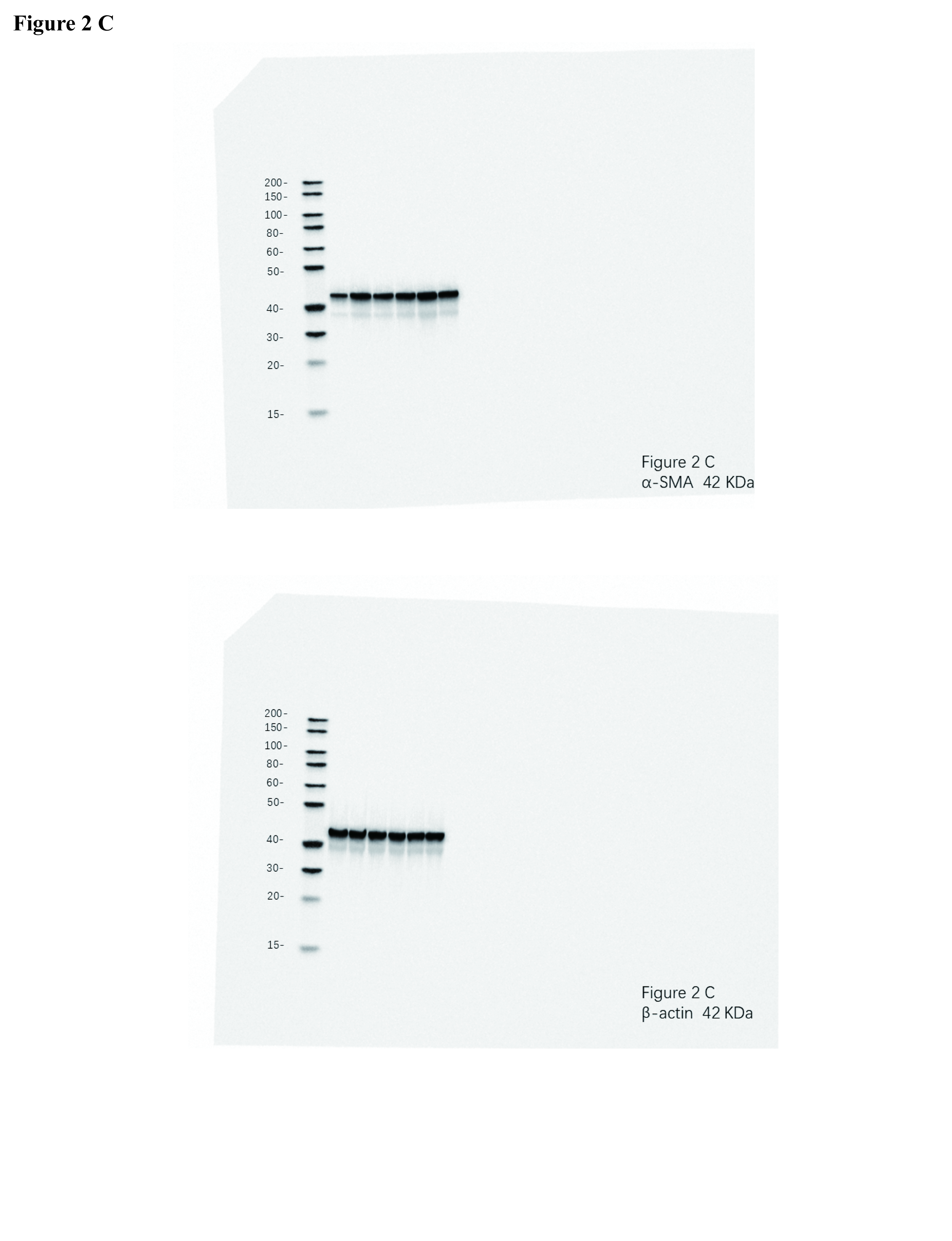

Supplement: Supplementary file 5 — all Figures Uncropped blots [file 41420_2025_2739_MOESM5_ESM.zip › blot-01.tif]

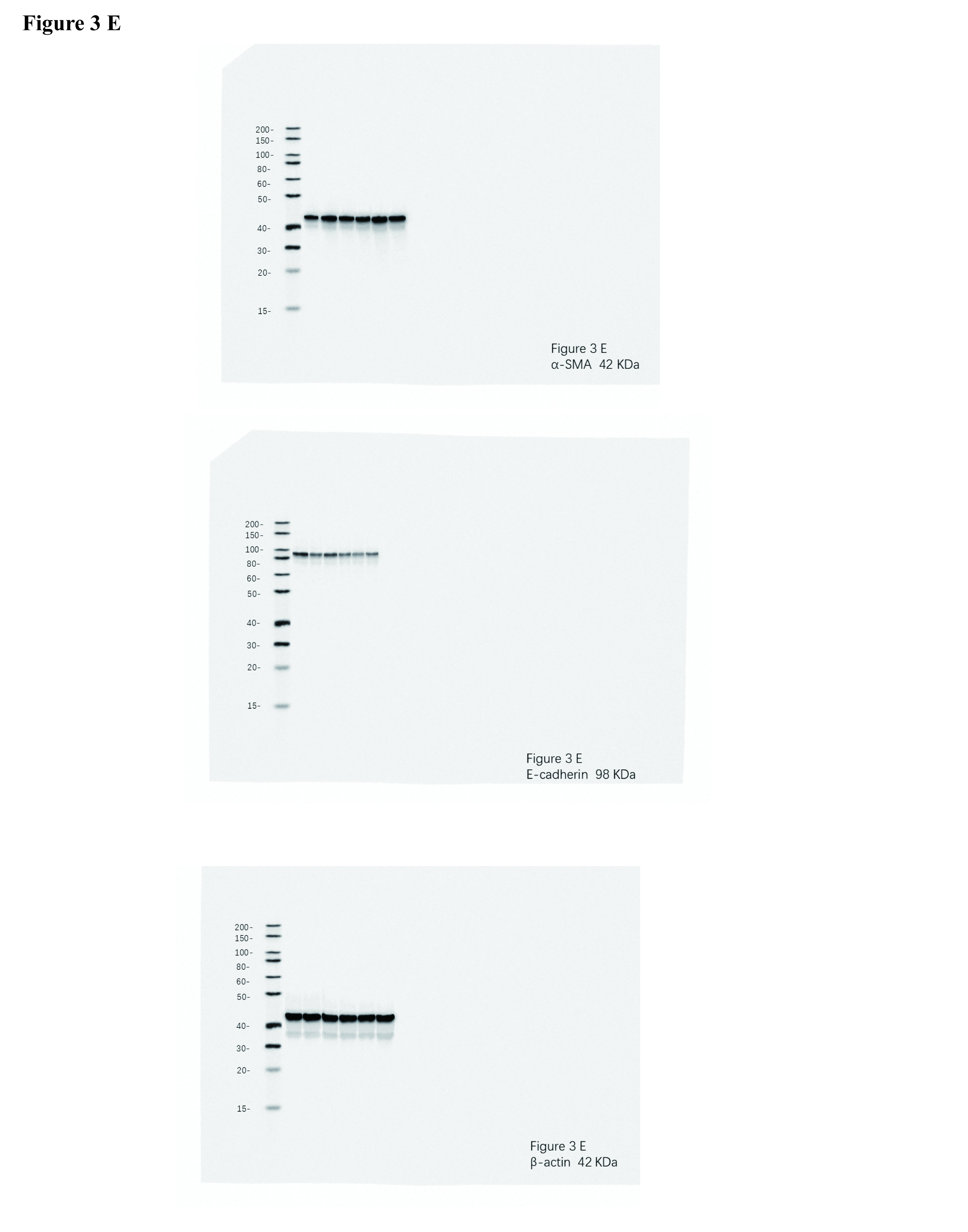

Supplement: Supplementary file 5 — all Figures Uncropped blots [file 41420_2025_2739_MOESM5_ESM.zip › blot-02.tif]

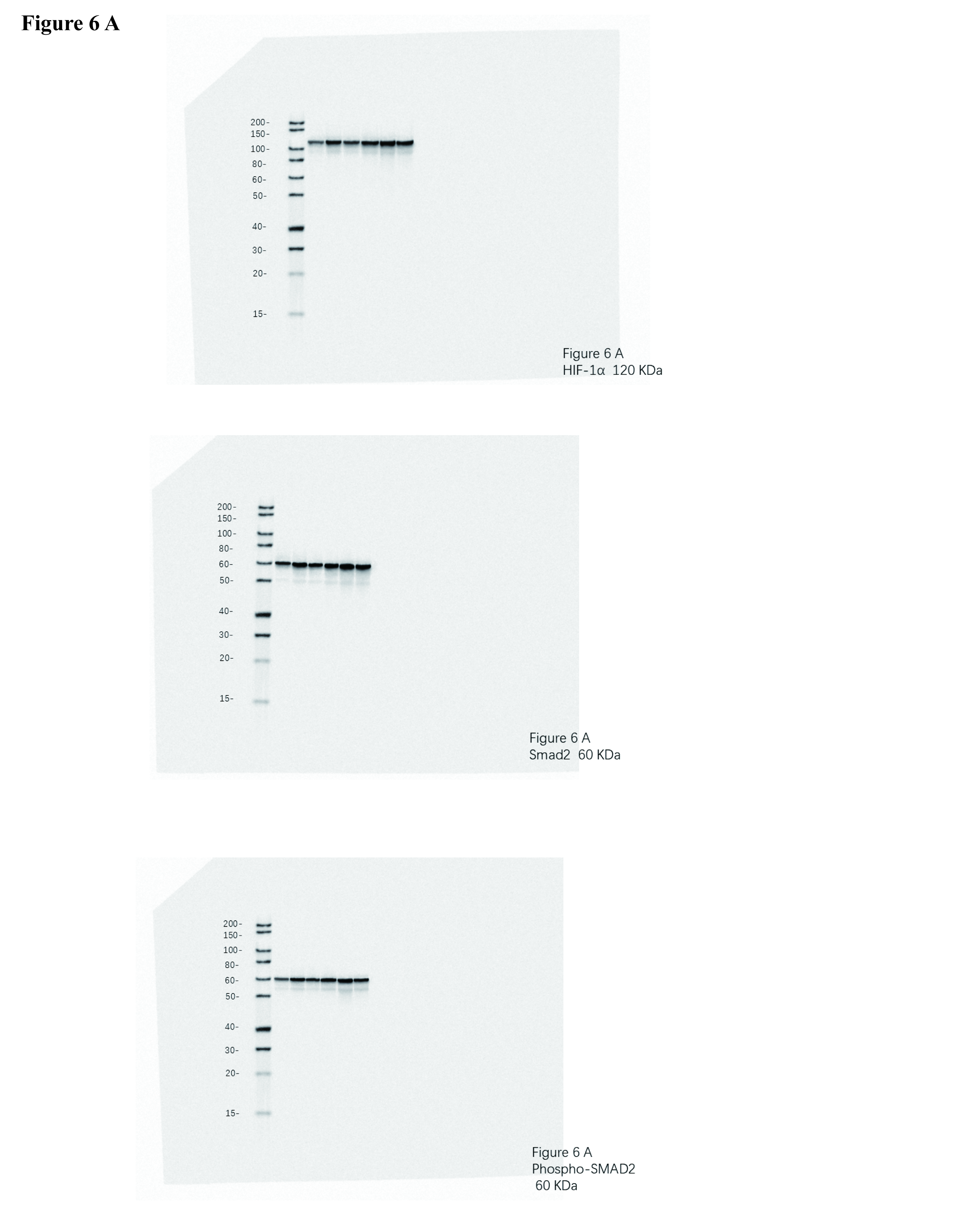

Supplement: Supplementary file 5 — all Figures Uncropped blots [file 41420_2025_2739_MOESM5_ESM.zip › blot-03.tif]

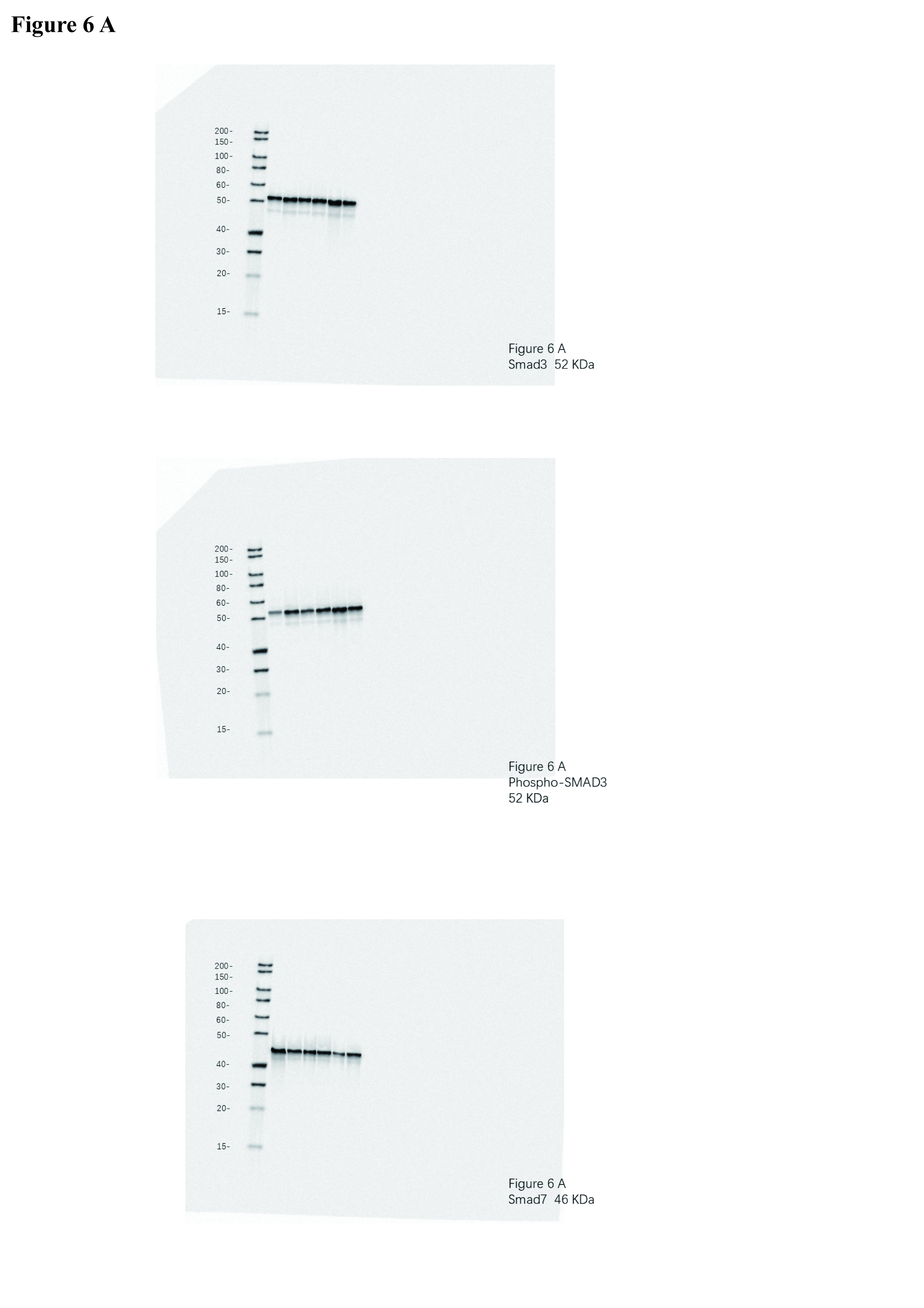

Supplement: Supplementary file 5 — all Figures Uncropped blots [file 41420_2025_2739_MOESM5_ESM.zip › blot-04.tif]

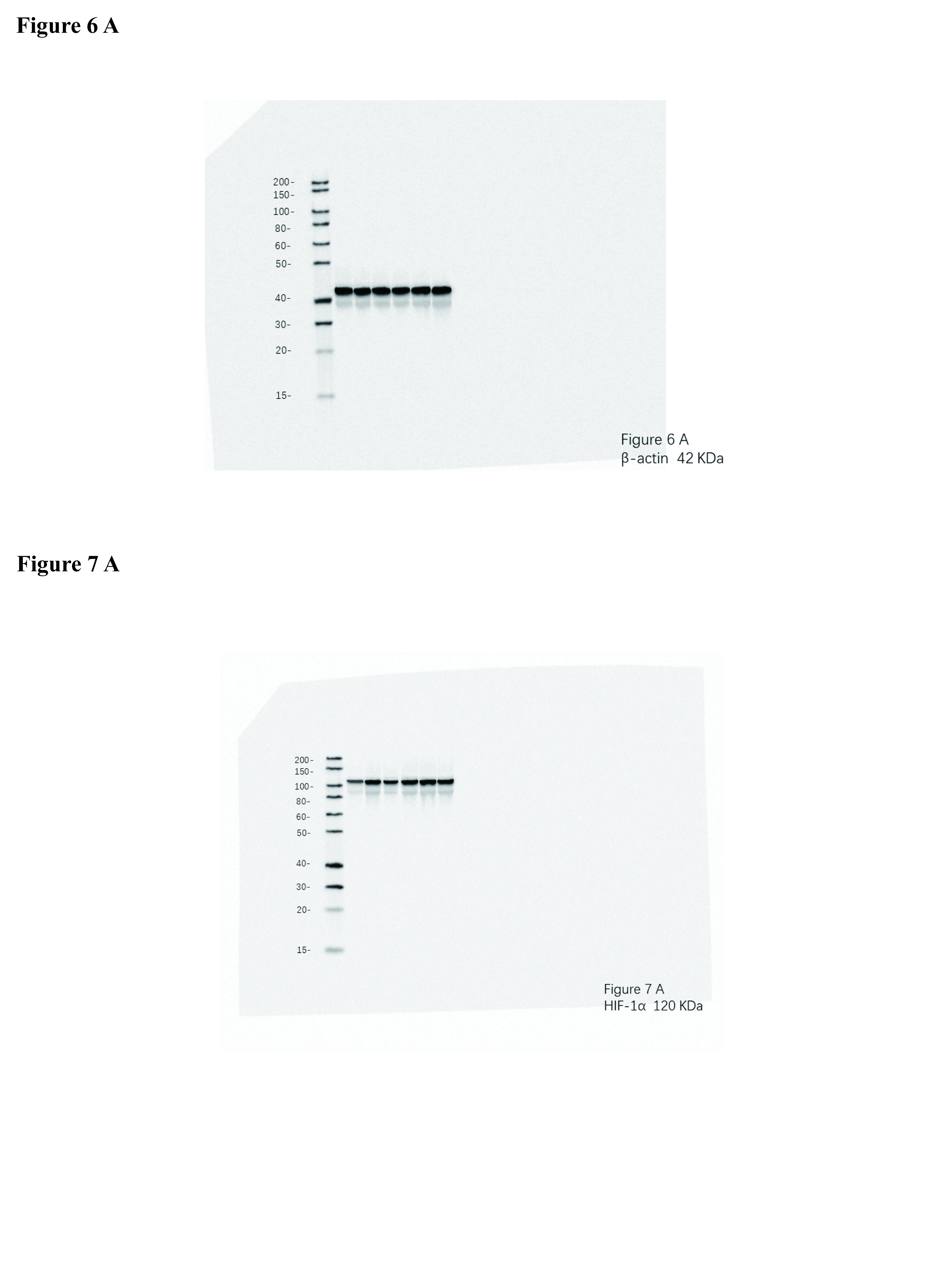

Supplement: Supplementary file 5 — all Figures Uncropped blots [file 41420_2025_2739_MOESM5_ESM.zip › blot-05.tif]

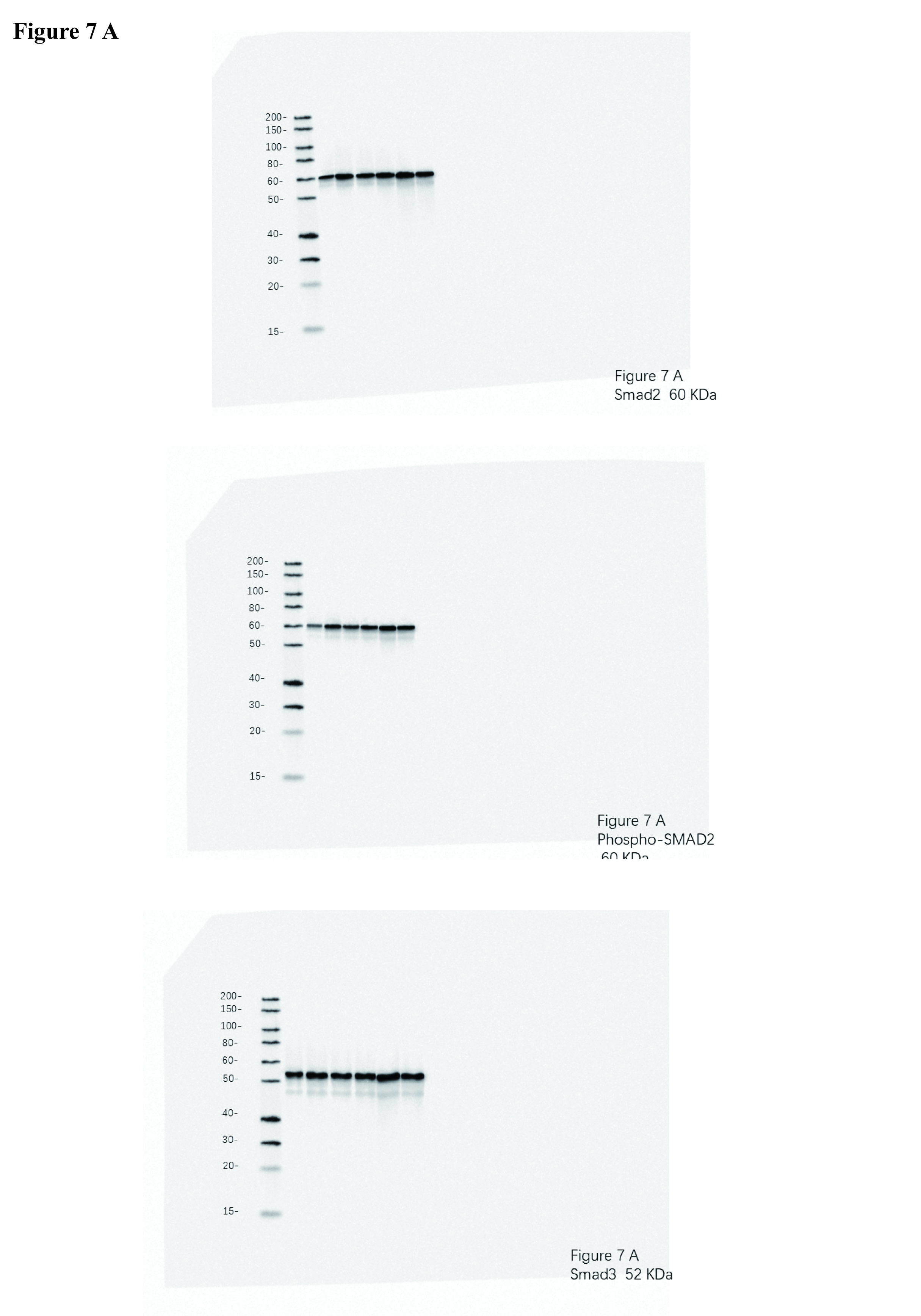

Supplement: Supplementary file 5 — all Figures Uncropped blots [file 41420_2025_2739_MOESM5_ESM.zip › blot-06.tif]

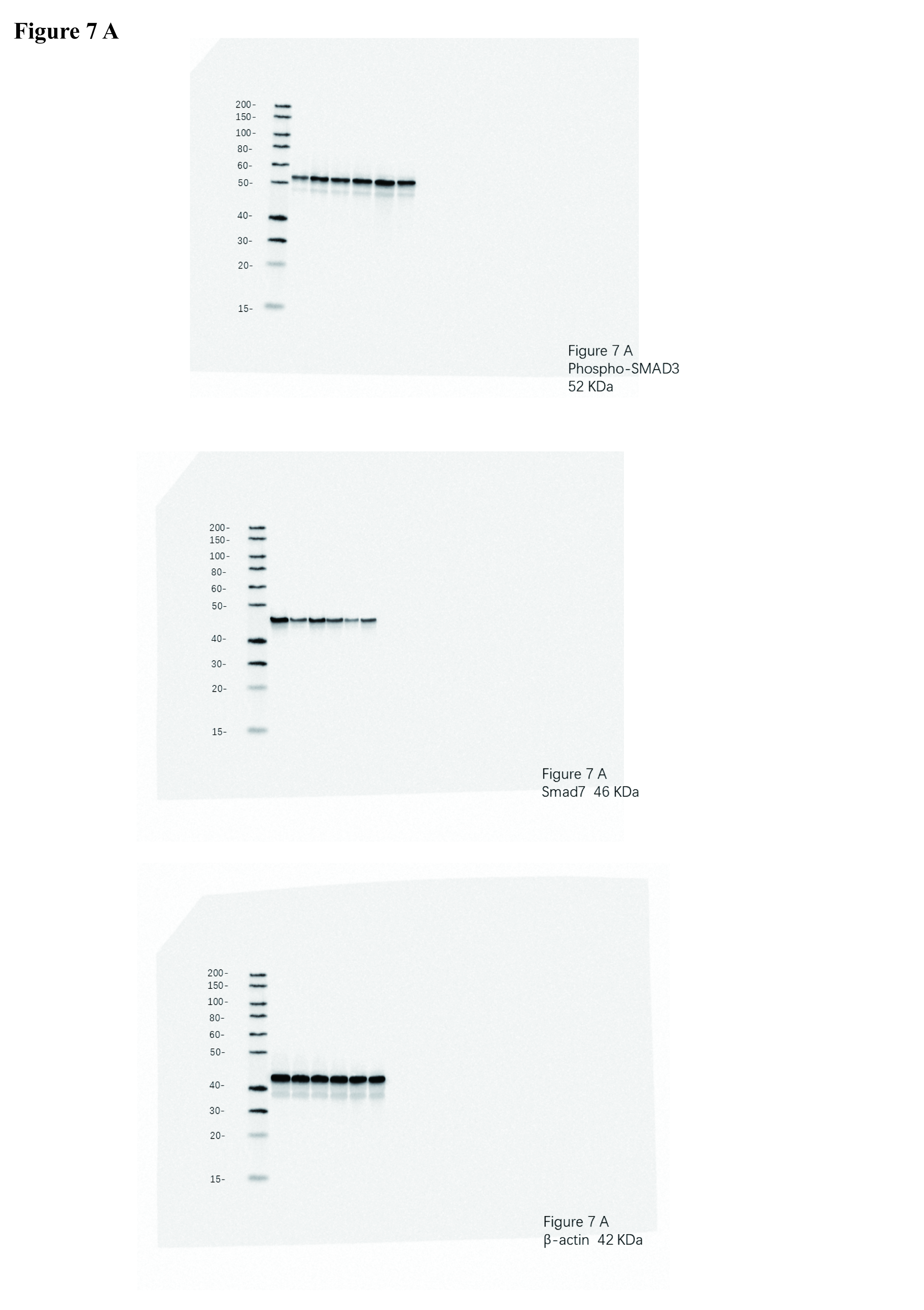

Supplement: Supplementary file 5 — all Figures Uncropped blots [file 41420_2025_2739_MOESM5_ESM.zip › blot-07.tif]

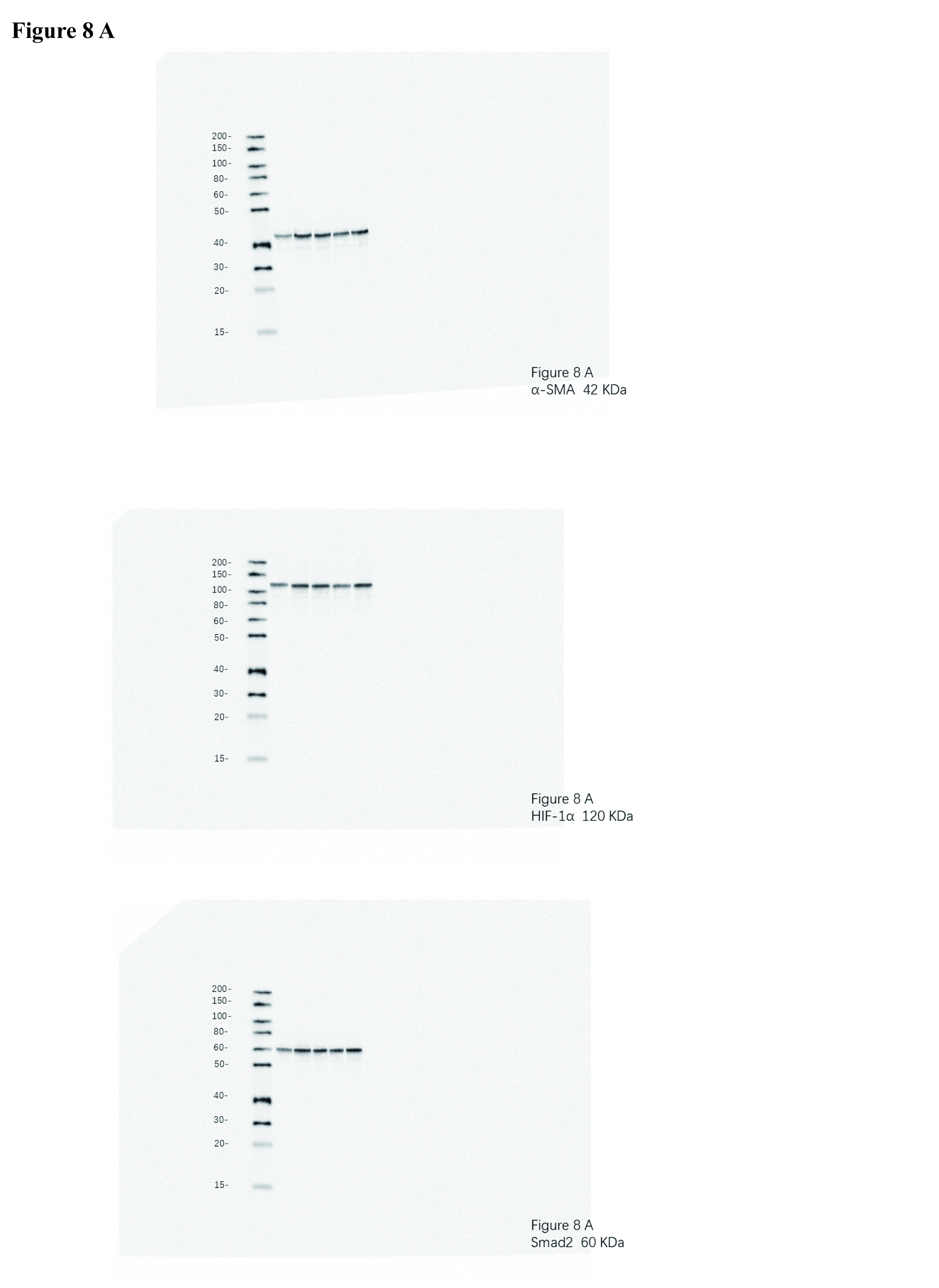

Supplement: Supplementary file 5 — all Figures Uncropped blots [file 41420_2025_2739_MOESM5_ESM.zip › blot-08.tif]

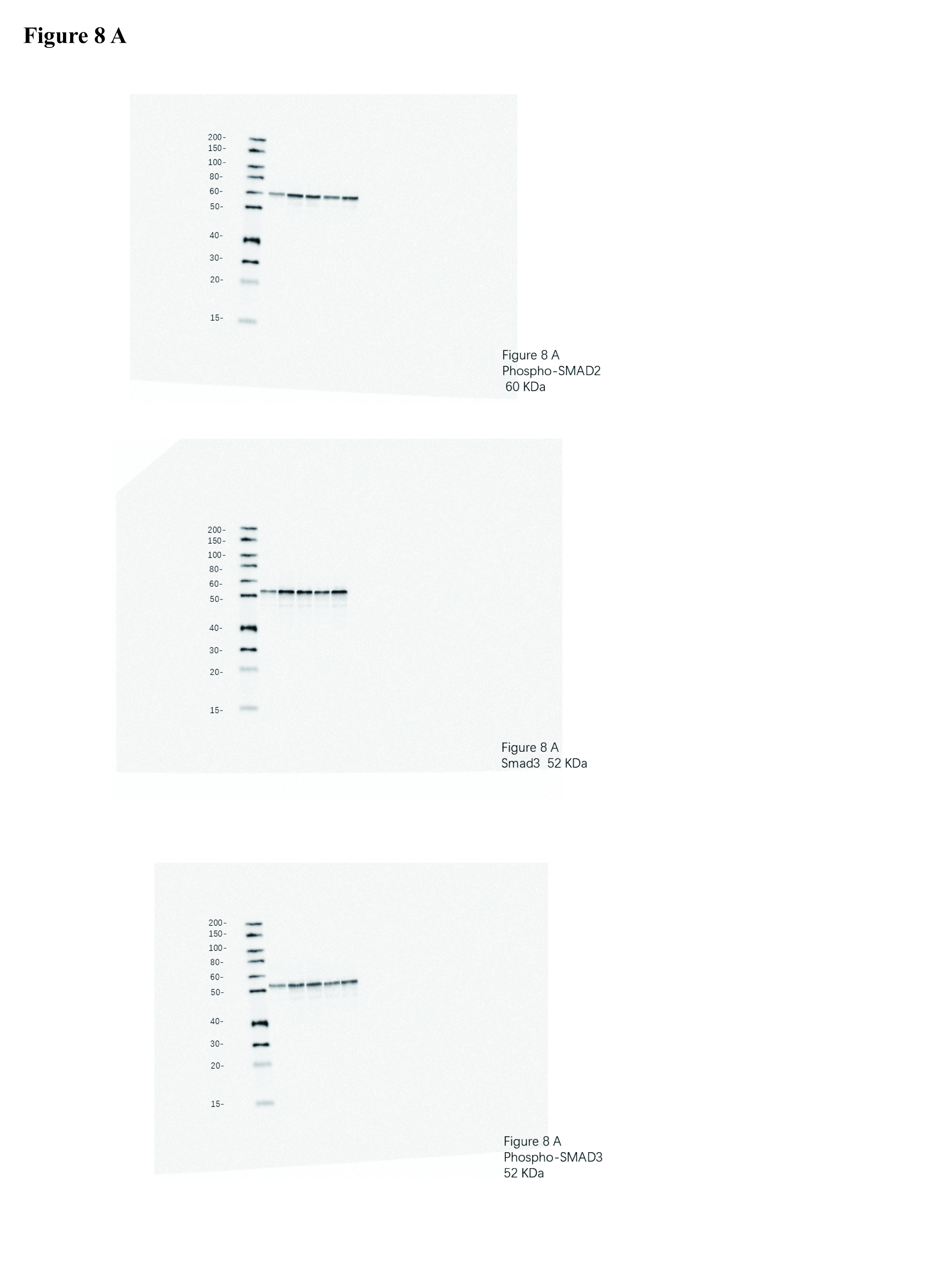

Supplement: Supplementary file 5 — all Figures Uncropped blots [file 41420_2025_2739_MOESM5_ESM.zip › blot-09.tif]

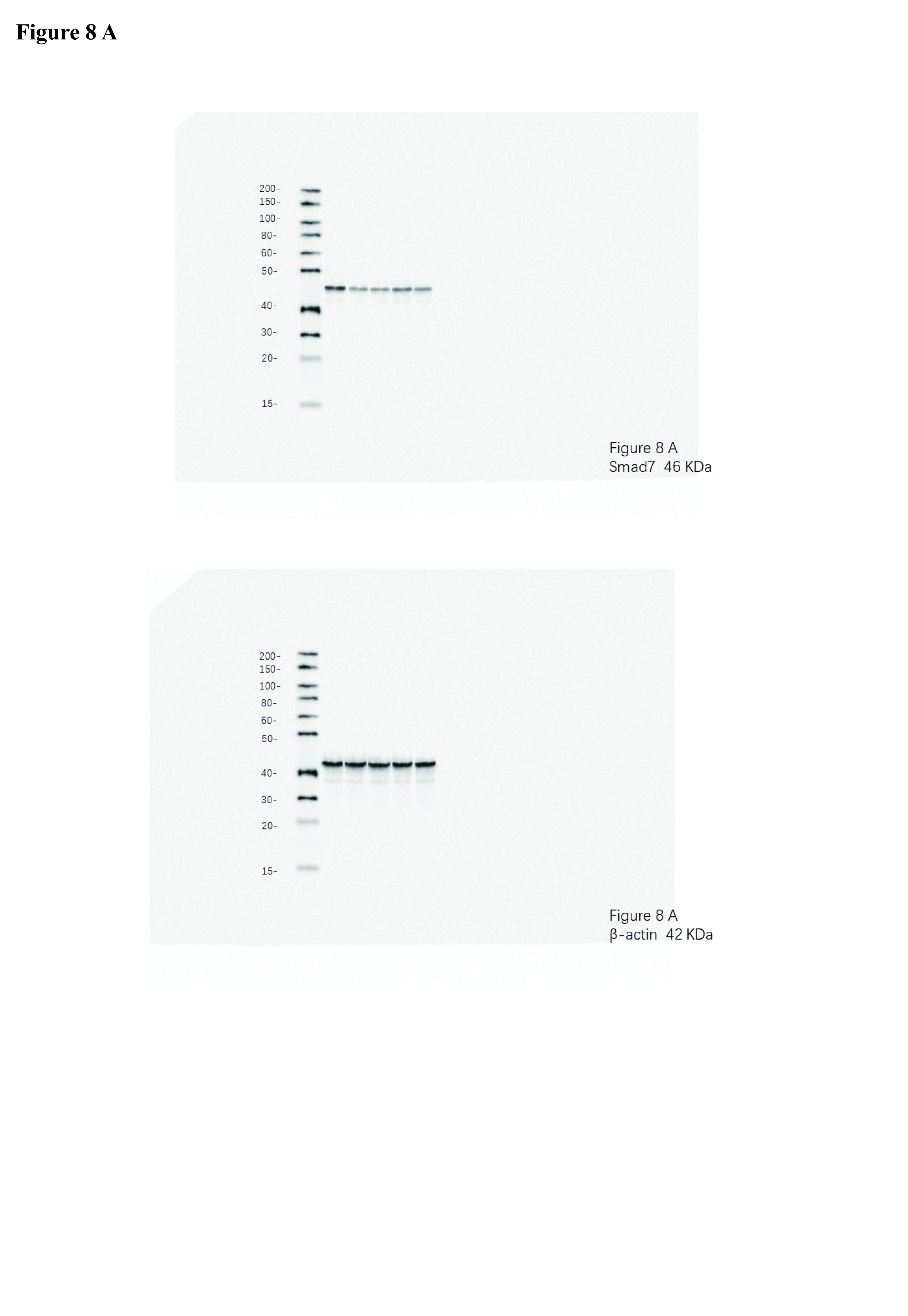

Supplement: Supplementary file 5 — all Figures Uncropped blots [file 41420_2025_2739_MOESM5_ESM.zip › blot-10.tif]

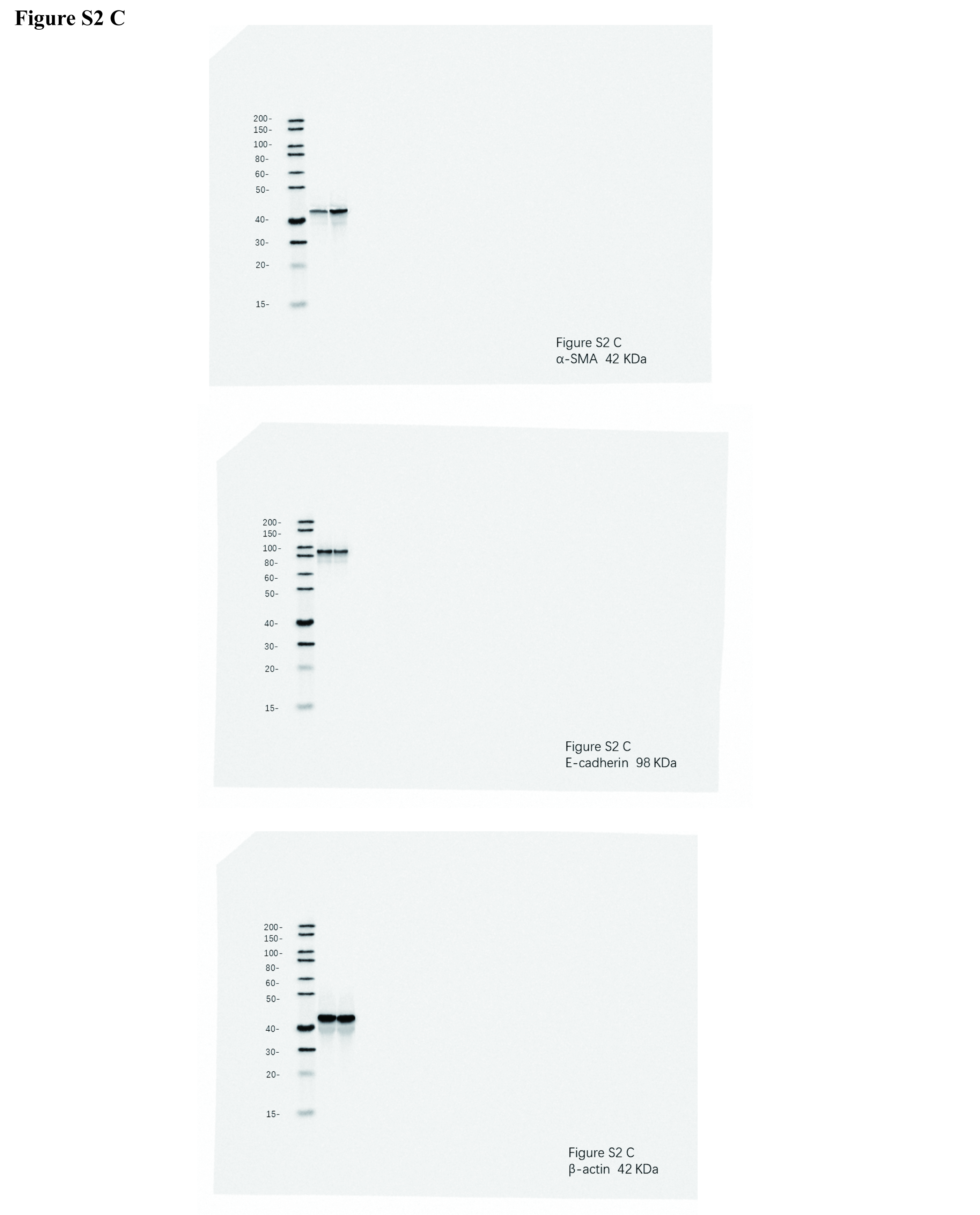

Supplement: Supplementary file 5 — all Figures Uncropped blots [file 41420_2025_2739_MOESM5_ESM.zip › blot-12.tif]
