# Supplementary material for: Exosomal miR-122-5p from tubular cells ameliorates renal interstitial fibrosis by regulating fibroblasts via HIF-1α
Source: Cell Death Discov. 2025 Oct 21;11:474. doi: 10.1038/s41420-025-02739-8 (PMC12540884; doi:10.1038/s41420-025-02739-8)
Supplement: Supplementary file 4 — Supplementary Figures Lengends [file 41420_2025_2739_MOESM4_ESM.docx]

**Supplementary data**

**
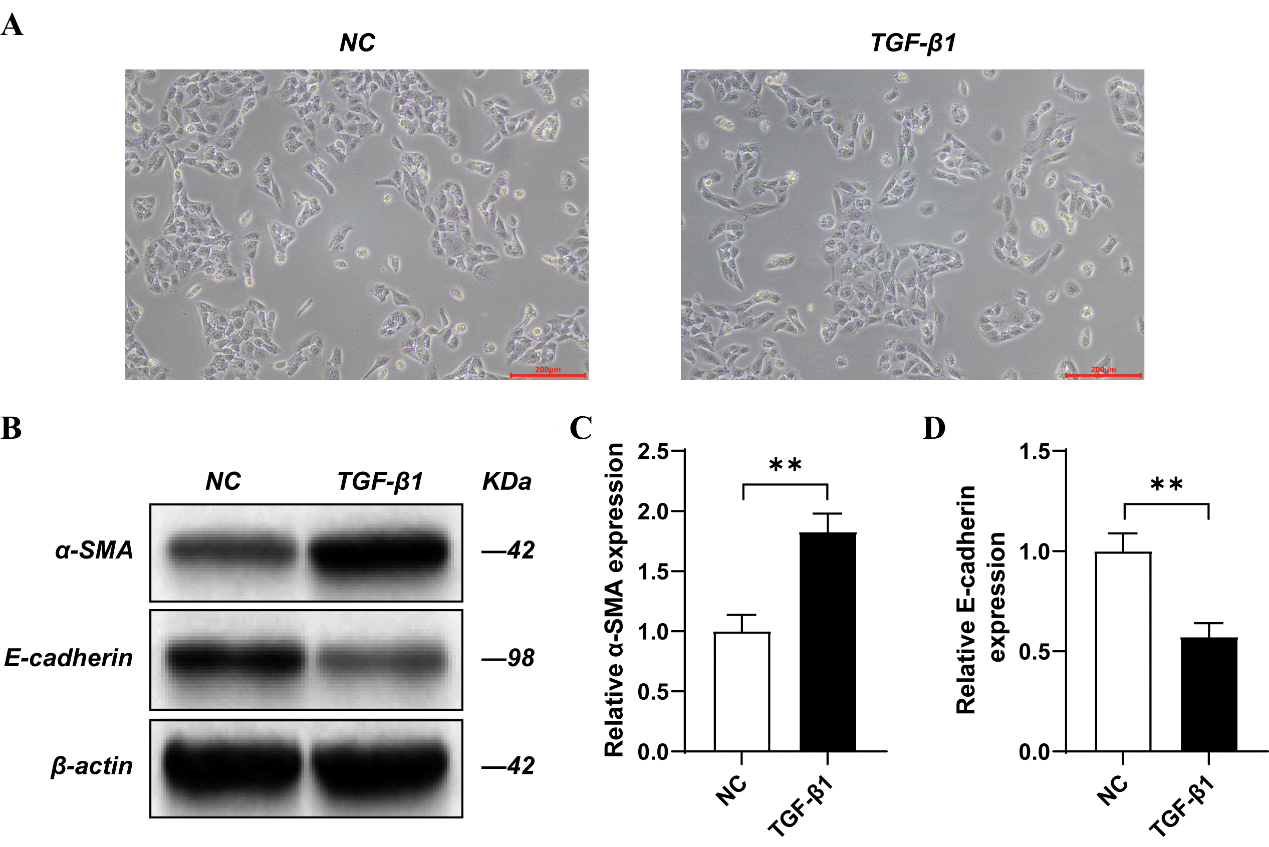
**

**Figure S1.** **TGF-β1 induced fibrosis in HK-2 cells.** A. HK-2 cells were incubated with or without TGF-β1 (10 ng/mL) for 24 h. Representative image from light microscopy (Bar = 200 μm); B. The expression levels of α-SMA and E-cadherin in HK-2 cells were detected by western blotting; C. Relative expression levels of α-SMA in HK-2 cells; D. Relative expression levels of E-cadherin in HK-2 cells. Results are shown as mean ± SD for three individual experiments. ^**^*P* < 0.01.


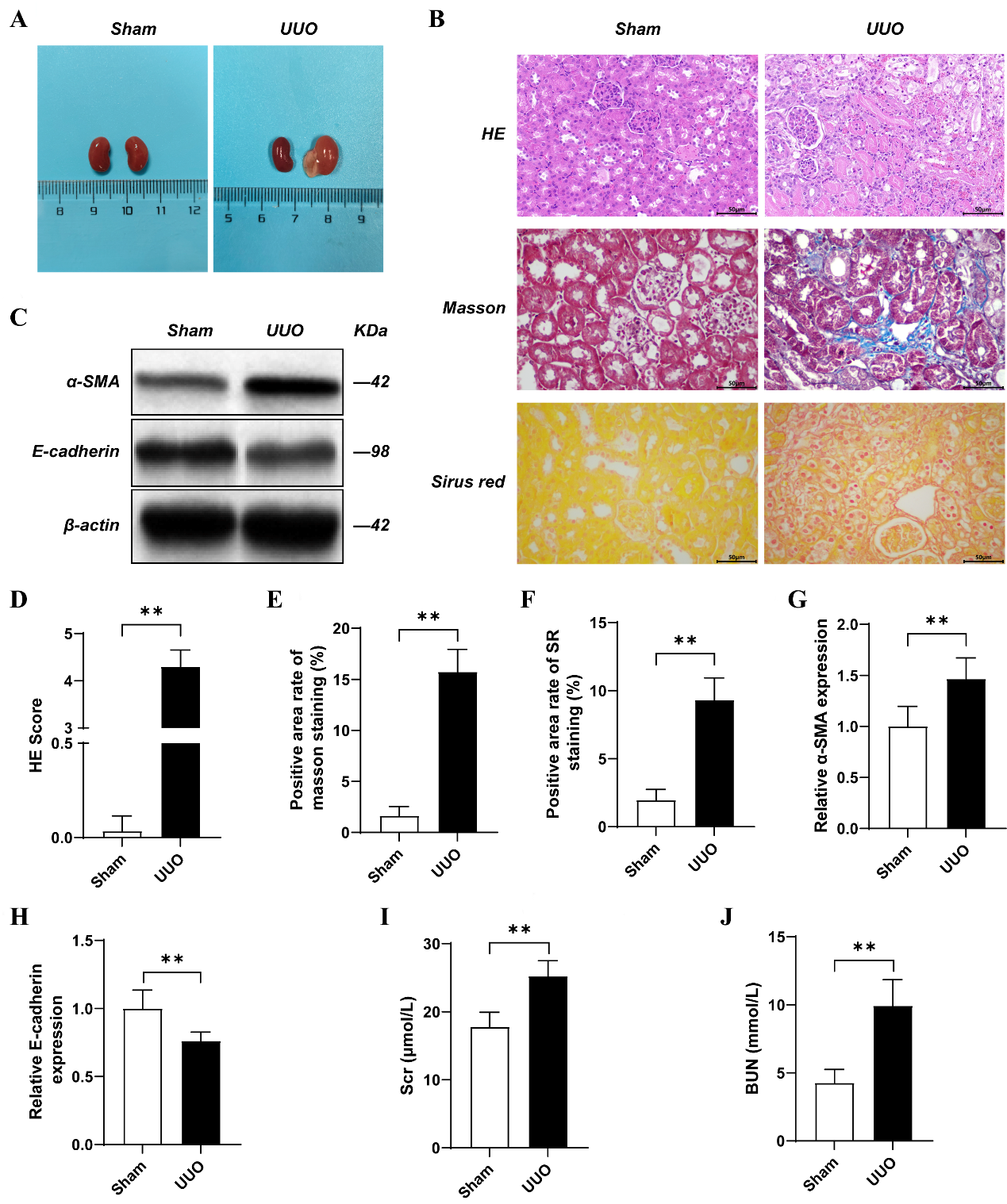


**Figure S2.** **Establishment of a mouse model of UUO.** A. Representative images of kidneys; B. Pathological results of HE, Masson’s and Sirius red staining; C. The expression levels of α-SMA and E-cadherin in renal tissues were detected by western blotting; D. HE staining statistics; E. Masson’s staining statistics; F. Sirius red staining statistics. G. Relative expression levels of α-SMA in renal tissues; H. Relative expression levels of E-cadherin in renal tissues; I. The levels of creatinine in mice serum were determined by a biochemical analyzer. J. The levels of blood urea nitrogen in mice serum were determined by a biochemical analyzer. Results are shown as the mean ± SD for six individual experiments. **P < 0.01.

**
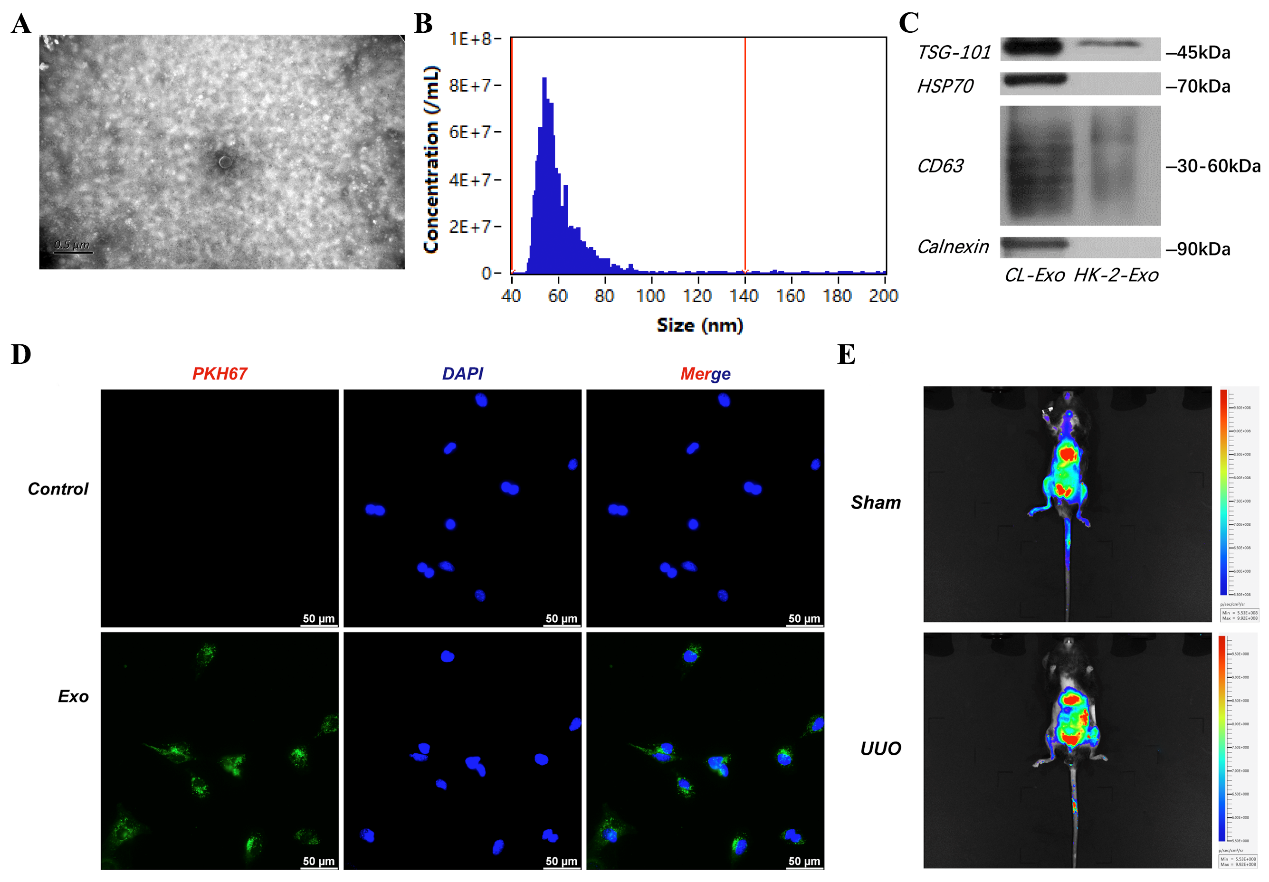
**

**Figure S3.** **Isolation, Characterization, Distribution, and Uptake of HK-2 Cell-Derived Exosomes in Tubular-Fibroblast Communication.** A. TEM image of exosomes isolated from HK-2 cells; B. NTA of exosomes from HK-2 cells; C. Representative western blot of TSG10, HSP7, CD63 and Calnexin as exosomal markers in exosomes from HK-2 cells. CL represents the cell lysate sample, used as a positive control in exosome Western blot identification. D. Representative images captured under a fluorescence microscope; E. The distribution of these exosomes was observed using an *in vivo* imaging system at 750 nm emission wavelength.
